# A macrophage-intestinal organoid co-culture model captures balanced epithelial homeostasis and inflammation

**DOI:** 10.1016/j.stemcr.2026.103045

**Published:** 2026-08-06

**Authors:** Lisa Tonini, Tae Baek Lee, Eunju Jang, Sungmoo Hong, Soobin Choi, Changhwan Ahn

**Affiliations:** 1 Veterinary Physiology Laboratory, College of Veterinary Medicine, Jeju National University, Jeju 63243, Republic of Korea; 2 Department of Veterinary Medicine and Veterinary Medical Research Institute, College of Veterinary Medicine, Jeju National University, Jeju 63243, Republic of Korea

**Keywords:** intestinal organoids, BMDM, co-culture model, intestinal stem cells, epithelial-immune crosstalk, inflammatory remodeling

## Abstract

Immune-epithelial interactions are essential for intestinal homeostasis, yet organoid monocultures lack immune components and fail to model how immune cells shape epithelial identity. Here, we establish a macrophage-organoid co-culture integrating bone marrow-derived macrophages (BMDMs) into the intestinal niche to create a controlled, immune-competent system. Under homeostatic conditions, the optimized 5k configuration of BMDMs preserves crypt-like architecture and maintains stem cell localization and secretory lineage organization. TNFα stimulation induces coordinated epithelial remodeling characterized by reduced canonical stem cell marker expression and density-dependent shifts in macrophage phenotype. Transcriptomic profiling confirms enrichment of immune-associated and epithelial stress pathways. Comparative analysis with a DSS-induced murine inflammation model reveals overlapping inflammatory and epithelial remodeling features, supporting physiological relevance. This platform provides a reproducible framework for studying epithelial-immune interactions and inflammatory remodeling under defined conditions, offering a controlled alternative to animal models.

## Highlights

• Intestinal organoid-macrophage co-culture preserves features of *in vivo* system
• Macrophage density shapes epithelial inflammatory responsiveness in co-culture
• TNFα exposure drives coordinated epithelial remodeling and revival stem cell
• Co-culture system recapitulates key epithelial features of DSS-induced IBD model

## Teaser

Tonini et al. establish a murine intestinal organoid-macrophage co-culture platform that preserves crypt architecture and stem cell identity under homeostatic conditions. Macrophage density shapes epithelial organization and inflammatory responsiveness. TNFα stimulation drives coordinated epithelial remodeling and revival stem cell transcriptional transition in a density-dependent manner, recapitulating key inflammatory and epithelial features of DSS-induced intestinal inflammation *in vivo*.

## Introduction

The intestinal epithelium constitutes a dynamic barrier ensuring host protection, nutrient absorption, and immune balance (Groschwitz and Hogan 2009; Kwon et al., 2020). Complete self-renewal every 3–5 days depends on finely tuned communication among epithelial, stromal, and immune cells within the mucosal microenvironment (van der Flier and Clevers 2009; Kabouridis et al., 2015; Obata and Pachnis 2016), essential for maintaining homeostasis and orchestrating inflammatory responses (Malijauskaite et al., 2021). Current efforts increasingly aim to reduce animal use by developing *in vitro* systems that faithfully reproduce intestinal complexity (Lee et al., 2020; Richter 2024).

Intestinal organoids derived from adult stem cells have emerged as a powerful *in vitro* platform to investigate epithelial dynamics and regeneration (Sato et al., 2009; Fujii et al., 2018), faithfully recapitulating crypt-villus architecture and key epithelial lineages (Pleguezuelos-Manzano et al., 2020; Qu et al., 2021). However, organoid monocultures lack physiologically relevant immune and stromal components fundamental for regulating epithelial turnover, coordinating inflammatory responses, and preserving barrier function (Taelman et al., 2022; Tonini and Ahn 2025). Current immune co-culture attempts often rely on supraphysiological cell densities or strong pro-inflammatory stimuli, leading to exaggerated cytokine production and epithelial disruption (Schreurs et al., 2021; Recaldin et al., 2024; Al-Qadami et al., 2025). Consequently, there remains a need for controlled, immune-competent co-culture systems that preserve epithelial integrity while enabling a more realistic representation of the intestinal microenvironment. Recent advances in organoid-macrophage co-culture systems have begun to address this gap (Kakni et al., 2022; Moraitis et al., 2025), yet controlled platforms incorporating primary macrophages at physiological densities with parallel *in vivo* validation remain limited. To address these limitations, we established a murine intestinal organoid co-culture incorporating bone marrow-derived macrophages (BMDMs). By integrating BMDMs directly into the epithelial niche, our system bridges the gap between organoid monocultures and the *in vivo* intestine, enabling physiologically relevant epithelial-immune communication. Testing two densities (1k and 5k BMDMs/well), we found that both conditions supported epithelial homeostasis, but 5k more consistently preserved crypt-like architecture. Co-cultures were exposed to TNFα (15 ng/mL) to model a controlled inflammatory environment, revealing inflammatory and epithelial remodeling features resembling aspects of intestinal inflammatory responses. Physiological relevance was further supported by comparative analysis with a Dextran Sulfate Sodium (DSS)-induced murine colitis model using histological, flow cytometric, and systemic inflammatory assessments. Collectively, this platform provides a biologically relevant framework for studying immune-associated epithelial remodeling and inflammatory responses in the intestine.

## Results

### Generation of immunocompetent intestinal organoid co-cultures

**Figure 1:**
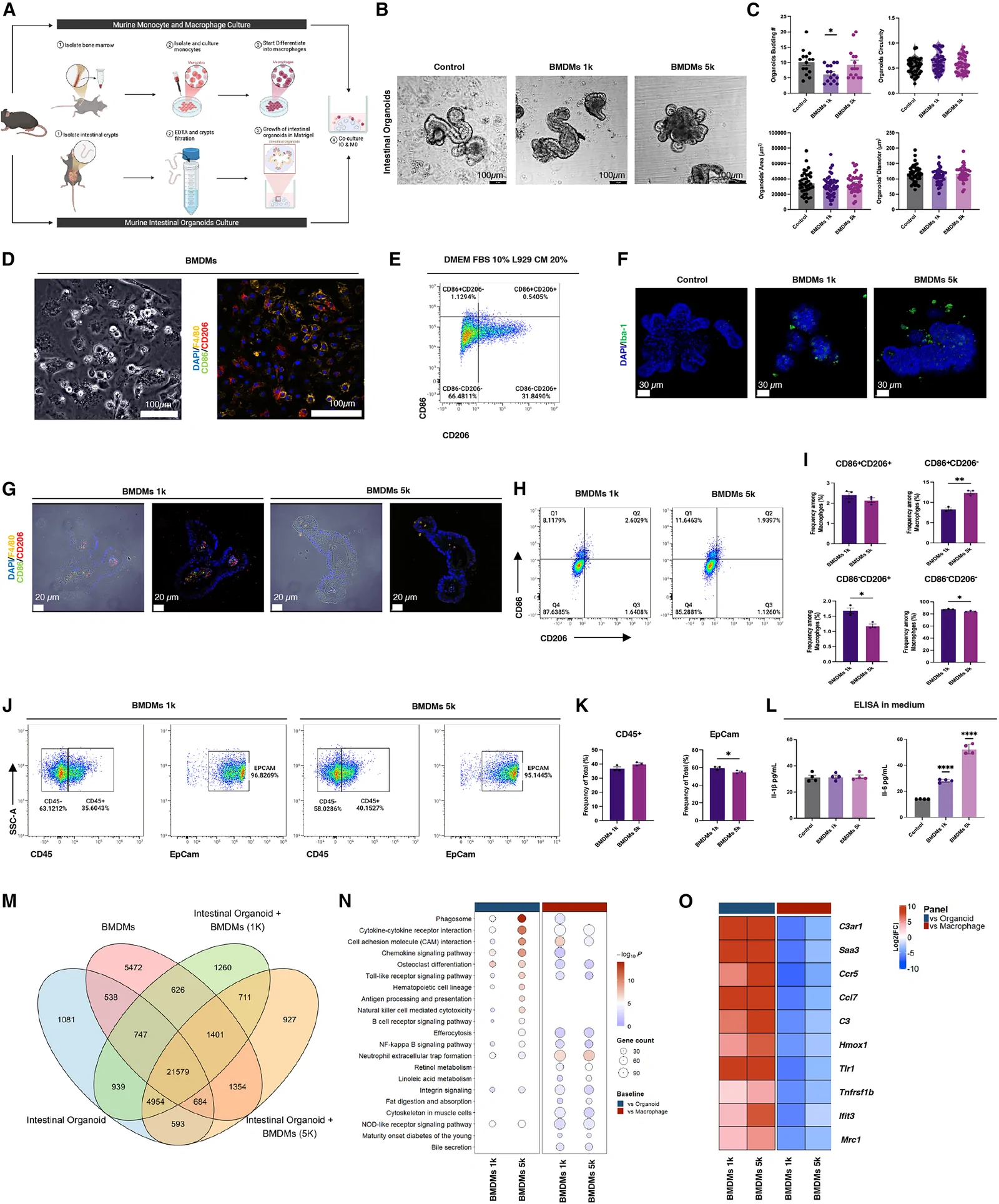
Establishment and characterization of a murine macrophage-intestinal organoid co-culture system (A) Experimental workflow schematic for BMDM isolation, differentiation, and co-culture with intestinal organoids. (B) Representative bright-field images of organoids cultured with control, 1k, or 5k BMDMs for 24 h. Scale bars, 100 μm. (C) Quantification of budding number, circularity, area, and diameter (*n* = 40 organoids/condition, 5 independent experiments). (D) Bright-field and immunofluorescence images of BMDMs in DMEM + 10% FBS + 20% L929 conditioned medium (CM), stained for DAPI, F4/80, CD86, and CD206. Scale bars, 100 μm. (E) Representative flow cytometry dot plot of CD86 and CD206 expression in L929 Conditioned Medium-differentiated BMDMs. (F) Immunofluorescence of co-cultured organoids stained for DAPI and Iba-1. Scale bars, 50 μm. (G) Immunofluorescence of co-cultured organoids stained for DAPI, F4/80, CD86, and CD206. Scale bars, 20 μm. Representative images from 5 independent experiments. (H) Flow cytometry dot plots of CD86/CD206 distribution within F4/80^+^CD45^+^ macrophages in 1k and 5k co-cultures. (I) Quantification of CD86^+^CD206^+^, CD86^+^CD206^−^, CD86^−^CD206^+^, and CD86^−^CD206^−^ macrophage subpopulations (*n* = 5 replicates/condition, 3 independent experiments). (J) Flow cytometry dot plots of CD45 and EpCam distribution in 1k and 5k co-cultures. (K) Quantification of CD45^+^ and EpCam^+^ proportions as frequency of total live cells (*n* = 5 replicates/condition, 3 independent experiments). (L) ELISA quantification of Il-1β and Il-6 in conditioned medium from control, 1k, and 5k conditions (*n* = 5 replicates/condition, 3 independent experiments). (M) Venn diagram of differentially expressed genes across organoid, BMDM, and co-culture transcriptomes (*n* = 4 samples/group). (N) Kyoto Encyclopedia of Genes and Genomes (KEGG) pathway enrichment dot plot comparing co-culture conditions against monoculture baselines (*n* = 4 samples/group). (O) Heatmap of selected inflammation-associated genes across co-culture conditions (*n* = 4 samples/group). Data: mean ± SEM. Statistics: one-way ANOVA with Tukey’s post hoc test. ^∗^*p* < 0.05, ^∗∗^*p* < 0.01, ^∗∗∗∗^*p <* 0.0001.

To establish a physiologically relevant immune-competent intestinal organoid model, we first evaluated multiple co-culture strategies for combining murine intestinal organoids with BMDMs. Because BMDMs were differentiated for 3 days in DMEM containing 10% fetal bovine serum (FBS) and 20% L929-conditioned medium, we initially investigated whether soluble immune-derived factors present in the macrophage culture medium could directly influence intestinal organoid morphology and behavior. For these experiments, day 3 intestinal organoids were exposed to increasing proportions of BMDM-conditioned medium mixed with intestinal organoid medium (Figures S1C and S1D). ELISA analysis confirmed elevated levels of Il-1β and Il-6 in BMDM-conditioned medium compared with control medium lacking macrophages, confirming the presence of macrophage-derived inflammatory signaling factors. Increasing concentrations of conditioned medium progressively altered organoid morphology, leading to increased circularity, suggesting reduced epithelial complexity. However, this approach exhibited substantial variability depending on conditioned medium concentration and lacked the dynamic bidirectional epithelial-immune interactions present *in vivo*. We next evaluated an indirect Transwell-based co-culture system (Figure S1E). Although this configuration physically separated epithelial and immune compartments, partial migration of BMDMs across the membrane was observed, limiting experimental consistency and compartmental control. We additionally tested co-culture following organoid subculture (Figures S1F and S1G); however, organoids subjected to passaging prior to macrophage exposure displayed a decrease in organoid diameter and increased circularity, likely reflecting transient stress associated with the dissociation process. Based on these observations, we established a direct-contact co-culture system using intact day 3 intestinal organoids cultured together with BMDMs for 24 h (Figure 1A). Preliminary titration experiments using high macrophage densities (10k–100k BMDMs/well; Figure S1H) consistently induced a spheroid-like organoid morphology associated with loss of epithelial budding. We, therefore, selected lower macrophage densities (1k and 5k BMDMs/well) for subsequent experiments (Figures 1B and 1C). Under these conditions, organoids generally maintained a budding phenotype without significant alterations in organoid area, diameter, or circularity. Interestingly, while the BMDM 1k condition showed a significant reduction in budding number compared with naive organoids, the 5k condition more effectively preserved crypt-like epithelial architecture and budding morphology (Figure 1D). These findings established 1k and 5k BMDM co-cultures as suitable conditions for subsequent functional characterization.

### Phenotypic characterization of BMDMs and validation of macrophage-organoid interactions

To characterize the immune component of the co-culture system, we first evaluated the phenotype of BMDMs differentiated using L929-conditioned medium. Because this differentiation strategy differs from conventional cytokine-based polarization protocols, macrophages were characterized by immunofluorescence staining for F4/80, CD86, and CD206 under both differentiation and co-culture conditions (Figures 1D and 1E; Figure S2A). As expected, supplementation with L929-conditioned medium promoted a substantially larger macrophage population, through CD206^+^ population, compared with DMEM containing 10% FBS alone, confirming efficient macrophage differentiation and survival support. Following differentiation, BMDMs were transferred into intestinal organoid medium (IntestiCult) during the 24-h co-culture phase based on the optimization experiments described above. This strategy was selected to avoid the morphological alterations induced by conditioned medium exposure while maintaining a controlled epithelial environment. To determine whether short-term exposure to IntestiCult medium altered macrophage identity, we further characterized BMDMs after 24 h culture in organoid medium using both immunofluorescence and flow cytometry analyses (Figures S2A–S2C). Overall macrophage proportions remained comparable between L929-conditioned medium and IntestiCult conditions. However, flow cytometry analysis revealed an increased proportion of CD206^+^ cells and decrease of double-negative population following short-term exposure to IntestiCult medium compared with L929-conditioned medium, indicating modest phenotypic changes associated with the organoid culture medium.

To further validate the physiological relevance of the system, we investigated the spatial relationship between macrophages and intestinal organoids. Immunofluorescence staining for Iba-1 demonstrated that BMDMs localized in close proximity to organoids and in several instances, infiltrated the epithelial structure itself (Figure 1E; Figure S2D). Histological sectioning of co-cultured organoids further confirmed macrophage migration within the organoid compartment, with macrophages frequently detected within the luminal region (Figure 1G; Figure S2E). Flow cytometry analyses (Figures 1H and 1I; Figures S3A–S3C) using F4/80, CD86, and CD206 demonstrated that the overall macrophage phenotype was largely maintained between the 1k and 5k co-culture conditions, although differences in specific macrophage subpopulations were observed. In particular, the 5k co-culture condition displayed increased proportions of CD86^+^ cells, accompanied by a relative reduction in CD206^+^ macrophages compared with the 1k condition, indicating density-dependent shifts in macrophage marker distribution within the co-culture system. To further assess epithelial-immune composition within the co-culture model, flow cytometry analysis was performed using CD45 and EpCAM markers to distinguish immune and epithelial cell populations, respectively (Figures 1J and 1K; Figure S3D). While the proportion of CD45^+^ immune cells remained relatively stable between the two co-culture conditions, the 5k condition exhibited a reduction in the EpCam^+^ epithelial population compared with the 1k condition. These observations suggest that increased macrophage density may influence epithelial cell composition and organization within the co-culture environment.

### BMDM co-culture promotes immune-associated transcriptional remodeling

To evaluate the functional impact of macrophage co-culture, cytokine secretion was quantified by ELISA after 24 h (Figure 1L). Organoids co-cultured with BMDMs showed no detectable Il-1β secretion but significantly increased Il-6 levels compared with monocultures, particularly in the 5k condition, indicating establishment of a cytokine-responsive environment without overt epithelial disruption. Bulk RNA sequencing on organoid monocultures, BMDM monocultures, and 1k and 5k co-culture conditions revealed distinct gene expression profiles compared with both monoculture baselines, with shared and unique transcriptional signatures across conditions (Figures 1M–1O). Pathway enrichment analysis identified significant upregulation of cytokine-cytokine receptor interaction, chemokine signaling, Toll-like receptor signaling, NF-κB signaling, phagosome, and cell adhesion molecule pathways, more prominently in the 5k condition (Figure 1N). Consistent with these findings, inflammation-associated genes including *C3ar1*, *Saa3*, *Ccr5*, *Ccl7*, *C3, Hmox1*, and *Tlr1* were enriched in co-culture conditions relative to organoid monocultures (Figure 1O), supporting the establishment of an immune-responsive microenvironment within the co-culture system.

### BMDM co-culture preserves crypt architecture and epithelial stem cell programs

**Figure 2:**
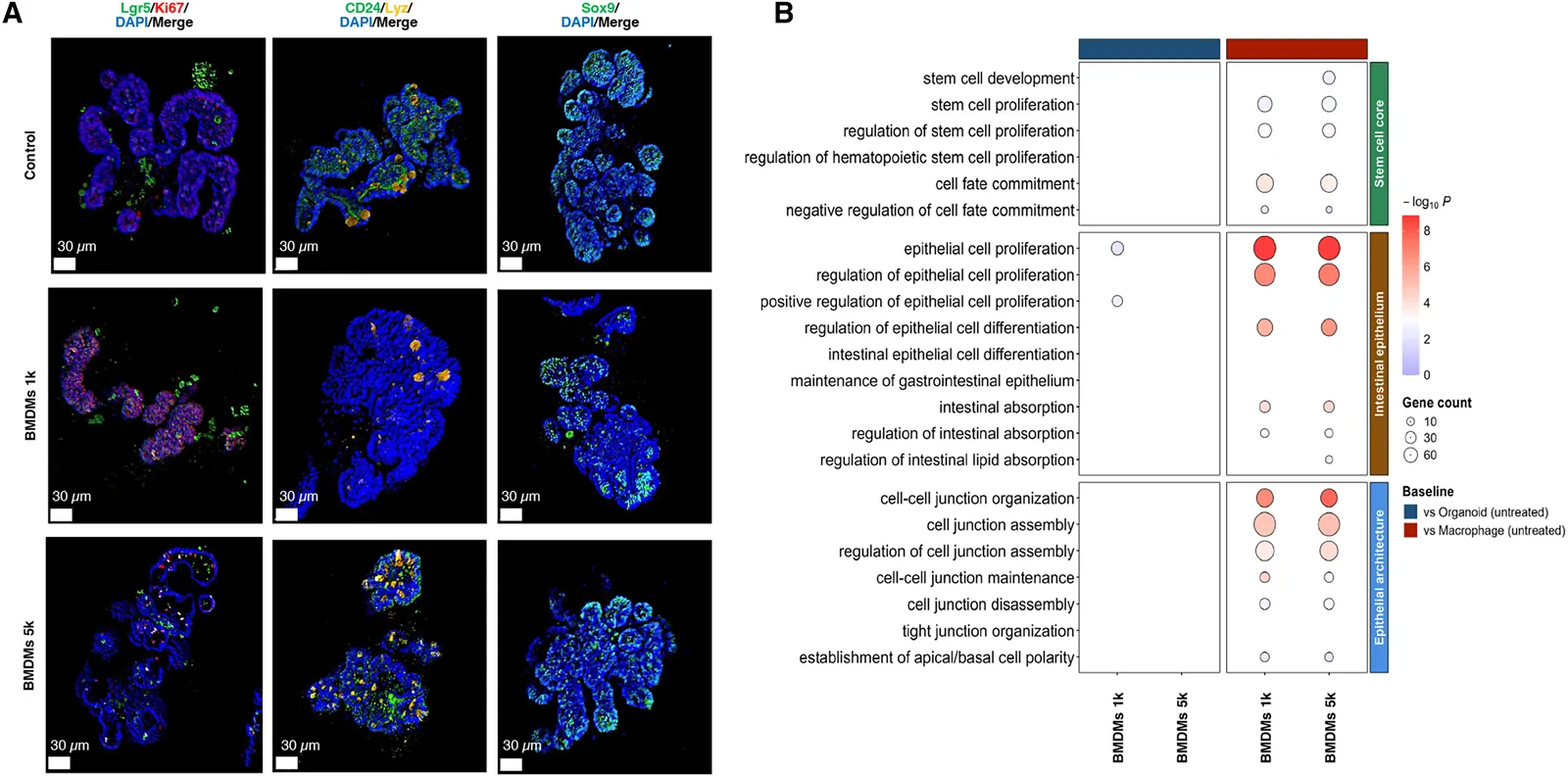
BMDM co-culture preserves crypt architecture and epithelial stem cell program (A) Representative immunofluorescence images of organoids under control, 1k, and 5k BMDM conditions, stained for Lgr5/Ki67/DAPI, CD24/Lyz/DAPI, and Sox9/DAPI. Scale bars, 30 μm. Representative images from 5 independent experiments. (B) GO biological process enrichment dot plot focusing on stem cell core, intestinal epithelium, and epithelial architecture pathways in 1k and 5k co-culture conditions versus organoid and macrophage monoculture baselines (*n* = 4 samples/group). Dot size represents gene count; color intensity indicates −log_10_*p* value. Data: mean ± SEM. Statistics: one-way ANOVA with Tukey’s post hoc test.

To assess whether BMDM co-culture preserves epithelial organization and stem cell compartment integrity, crypt morphology and niche-associated markers were analyzed by immunofluorescence (Figures 2A; Figures S4A–S4C). Lgr5^+^ intestinal stem cells remained localized at the crypt base across all conditions, with Ki67^+^ proliferative activity and overall epithelial architecture preserved. Paneth cell organization showed density-dependent differences—CD24 staining appeared partially disorganized in the 1k condition, whereas the 5k condition retained crypt-associated CD24 and Lyz distribution more closely resembling controls. Sox9 expression remained stable across all groups, supporting preservation of epithelial progenitor identity.

Bulk RNA sequencing analysis focused on stem cell maintenance, epithelial organization, and intestinal differentiation pathways (Figure 2B; Figures S4D and S4E). GO enrichment analysis revealed that both co-culture conditions retained pathways associated with stem cell development, epithelial differentiation, and barrier organization relative to macrophage monocultures, with only modest differences compared with organoid monocultures. Heatmap analysis confirmed overall preservation of intestinal stem cell markers including *Lgr5, Ascl2, Olfm4, Smoc2*, and *Prom1*, together with maintained WNT-, YAP-, and Notch-related signaling components and broad preservation of epithelial lineage markers across co-culture groups (Figures S4D and S4E). Collectively, these findings indicate that macrophage co-culture maintains epithelial organization and stem cell-associated transcriptional identity while preserving a structured crypt-like epithelial environment.

### TNFα exposure induces coordinated inflammatory remodeling in macrophage-organoid co-cultures

**Figure 3:**
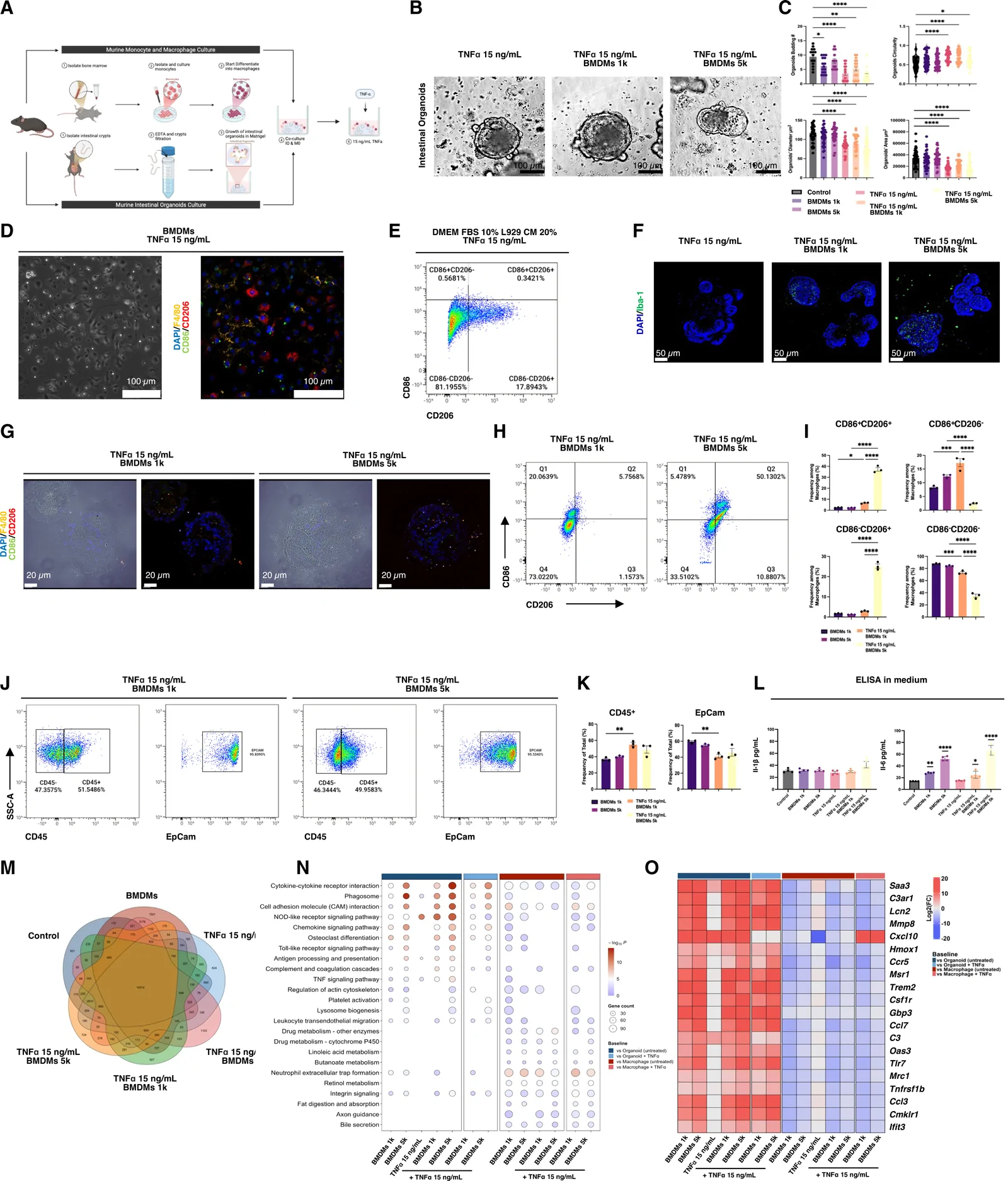
TNF exposure induces coordinated inflammatory remodeling in macrophage-organoid co-cultures (A) Experimental workflow schematic for TNFα stimulation in macrophage-organoid co-culture conditions. (B) Representative bright-field images of organoids exposed to TNFα (15 ng/mL) alone or with 1k or 5k BMDMs for 24 h. Scale bars, 100 μm. (C) Quantification of budding number, circularity, area, and diameter across TNFα-treated conditions (*n* = 40 organoids/condition, 5 independent experiments). (D) Bright-field and immunofluorescence images of BMDMs stimulated with TNFα (15 ng/mL) in DMEM + 10% FBS + 20% L929 CM, stained for DAPI, F4/80, CD86, and CD206. Scale bars, 100 μm. (E) Flow cytometry dot plot of CD86 and CD206 in TNFα-stimulated LCM-differentiated BMDMs. (F) Immunofluorescence of TNFα-treated co-cultured organoids stained for DAPI and Iba-1. Scale bars, 50 μm. (G) Immunofluorescence of TNFα-treated co-cultured organoids stained for DAPI, F4/80, CD86, and CD206. Scale bars, 20 μm. Representative images from 5 independent experiments. (H) Flow cytometry dot plots of CD86/CD206 distribution within F4/80^+^CD45^+^ macrophages in TNFα-treated 1k and 5k co-cultures. (I) Quantification of macrophage subpopulations in TNFα-treated conditions (*n* = 5 replicates/condition, 3 independent experiments). (J) Flow cytometry dot plots of CD45 and EpCam distribution in TNFα-treated co-cultures. (K) Quantification of CD45^+^ and EpCam^+^ proportions in TNFα-treated conditions (*n* = 5 replicates/condition, 3 independent experiments). (L) ELISA quantification of Il-1β and Il-6 in TNFα-treated conditioned medium (*n* = 5 replicates/condition, 3 independent experiments). (M) Venn diagram of differentially expressed genes across untreated and TNFα-treated monoculture and co-culture transcriptomes (*n* = 4 samples/group). (N) KEGG pathway enrichment dot plot comparing TNFα-treated co-cultures against untreated monoculture baselines (*n* = 4 samples/group). (O) Heatmap of selected inflammation-associated genes in TNFα-treated co-cultures (*n* = 4 samples/group). Data: mean ± SEM. Statistics: one-way ANOVA with Tukey’s post hoc test. ^∗^*p* < 0.05, ^∗∗^*p* < 0.01, ^∗∗∗^*p* < 0.001, ^∗∗∗∗^*p <* 0.0001.

To evaluate the response of the co-culture system under inflammatory conditions, intestinal organoids were exposed to TNFα following 3 days of organoid growth with simultaneous introduction of BMDMs (Figure 3A). Morphological analysis revealed that TNFα exposure induced a shift toward a more spheroid-like phenotype in both organoid monocultures and co-culture conditions, characterized by increased circularity together with reduced budding number, organoid area, and diameter compared with untreated controls (Figures 3B and 3C). We next characterized macrophage phenotypes following TNFα exposure under both L929-conditioned medium and IntestiCult conditions using immunofluorescence and flow cytometry analyses. TNFα stimulation induced a redistribution of macrophage phenotypes, characterized by an expansion of the double-negative (CD86^−^/CD206^−^) population and a relative shift toward CD206^+^ cells, suggesting that short-term TNFα exposure promotes a transitional rather than classically activated macrophage state under these culture conditions (Figures 3D and 3E; Figures S2A–S2C). To further assess epithelial-immune interactions during inflammatory challenge, TNFα-treated co-cultures were analyzed by Iba-1 immunofluorescence, histological sectioning, and flow cytometry (Figures 3F–3I; Figures S5A–S5D), as done for the co-culture condition without exposure. Similar to basal conditions, macrophages remained localized in close proximity to organoids and were frequently detected within the organoid compartment. Phenotypic characterization demonstrated density-dependent differences in macrophage marker distribution following TNFα exposure. In the 1k co-culture condition, macrophage phenotype remained largely comparable to untreated basal conditions. In contrast, the 5k condition exhibited a shift toward CD206-associated populations, characterized by increased proportions of CD86^+^/CD206^+^ double-positive and CD206^+^ single-positive macrophages, accompanied by a reduction in CD86^+^ single-positive and double-negative populations. Flow cytometry analysis of CD45 and EpCam populations further demonstrated reduced proportions of EpCam^+^ epithelial cells together with increased CD45^+^ immune populations in TNFα-treated co-cultures compared with untreated conditions (Figure 3J and 3K; Figures S5C and S5D). These findings are consistent with inflammatory remodeling of the epithelial compartment accompanied by increased immune-associated cellular representation within the co-culture system.

### BMDM co-culture modulates inflammatory signaling and transcriptional responses following TNFα exposure

Following TNFα stimulation, Il-1β levels remained low across all groups whereas Il-6 secretion was markedly increased in macrophage-containing co-cultures, with the strongest production in the 5k condition, indicating macrophages as the primary contributor to cytokine responsiveness (Figure 3L). Bulk RNA sequencing across untreated and TNFα-treated monoculture and co-culture conditions revealed distinct inflammatory transcriptional signatures in TNFα-treated co-cultures compared with untreated controls (Figures 3M–3O). Pathway enrichment analysis identified significant enrichment of cytokine-cytokine receptor interaction, chemokine signaling, Toll-like receptor signaling, TNF signaling, NOD-like receptor signaling, complement-associated pathways, and leukocyte transendothelial migration, particularly in the 5k condition (Figure 3N). Heatmap analysis confirmed enrichment of inflammation-associated genes including *Saa3*, *C3ar1*, *Lcn2*, *Mmp8*, *Cxcl10*, *Hmox1*, *Ccr5*, *Trem2*, *Csf1r*, *Ccl7*, *C3*, *Tlr7*, and *Ifit3* following TNFα exposure (Figure 3O), supporting establishment of a coordinated density-dependent inflammatory epithelial-immune transcriptional program. These transcriptional changes were accompanied by the morphological shift toward increased organoid circularity observed under TNFα exposure, consistent with an epithelial stress and remodeling response rather than complete epithelial collapse.

### TNFα exposure induces epithelial remodeling in macrophage-organoid co-cultures

**Figure 4:**
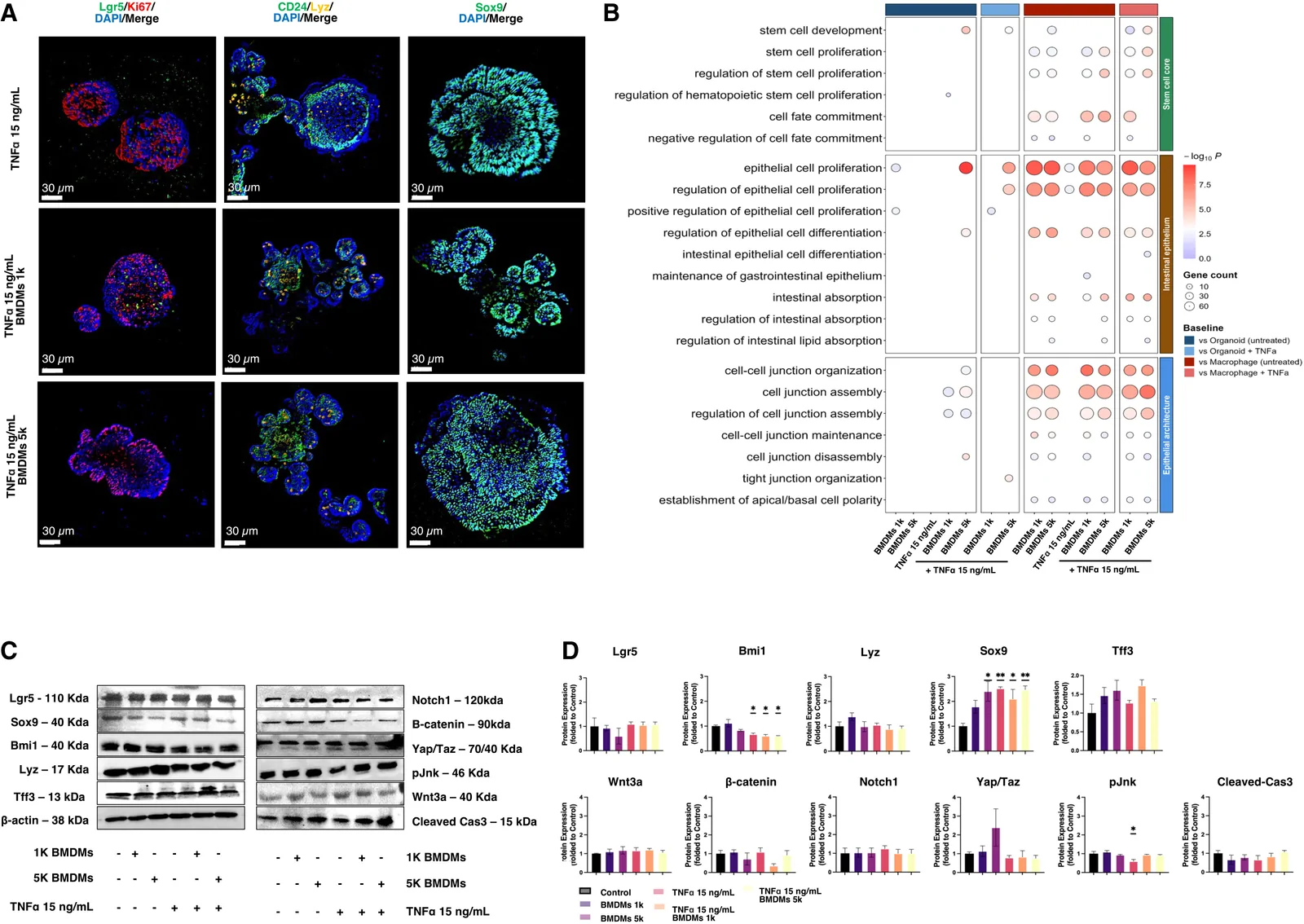
TNF exposure drives epithelial remodeling and stem cell transcriptional reprogramming in macrophage-organoid co-cultures (A) Representative immunofluorescence images of organoids exposed to TNFα (15 ng/mL) alone or with 1k or 5k BMDMs, stained for Lgr5/Ki67/DAPI, CD24/Lyz/DAPI, and Sox9/DAPI. Scale bars, 30 μm. Representative images from 5 independent experiments. (B) GO biological process enrichment dot plot focusing on stem cell core, intestinal epithelium, and epithelial architecture pathways in TNFα-treated 1k and 5k co-cultures versus untreated and TNFα-treated monoculture baselines (*n* = 4 samples/group). Dot size represents gene count; color intensity indicates −log_10_*p* value. (C) Representative western blot of Lgr5, Sox9, Bmi1, Lyz, Tff3, Notch1, β-catenin, Yap/Taz, Wnt3a, pJnk, and cleaved caspase-3 across all conditions. β-actin: loading control. (D) Quantification of protein expression normalized to β-actin and expressed relative to control (*n* = 3 independent experiments). Data: mean ± SEM. Statistics: one-way ANOVA with Tukey’s post hoc test. ^∗^*p* < 0.05, ^∗∗^*p* < 0.01.

To evaluate the effects of inflammatory stimulation on epithelial organization and stem cell-associated compartments, we analyzed intestinal organoids following TNFα exposure by immunofluorescence staining for Lgr5, Ki67, CD24, Lyz, and Sox9 (Figure 4A; Figures S6A–S6C). TNFα treatment induced marked morphological remodeling across all experimental groups, accompanied by a reduction of Lgr5^+^ stem cell staining, particularly in the 5k BMDM co-culture condition, consistent with disruption of crypt-like architecture under inflammatory stress. In parallel, a qualitative increase in Ki67^+^ proliferative cells was observed following TNFα exposure across both monoculture and co-culture conditions, indicating activation of epithelial proliferative responses. Despite these inflammatory changes, CD24 and Lyz staining remained detectable and largely retained within crypt-associated regions in co-culture conditions, suggesting partial preservation of Paneth cell-associated epithelial organization during inflammatory challenge. Similarly, Sox9 expression remained broadly maintained across TNFα-treated groups, supporting persistence of progenitor-associated epithelial populations during tissue remodeling.

### TNFα exposure drives stem cell and epithelial transcriptional reprogramming in co-cultures

To further characterize transcriptional remodeling induced by TNFα exposure, we performed bulk RNA sequencing analysis focusing on stem cell regulation, epithelial organization, and lineage identity. This analysis revealed that TNFα-treated co-cultures, particularly the 5k condition, displayed increased enrichment of stem cell development, epithelial proliferation, and cell junction organization pathways compared with monocultures, suggesting activation of coordinated epithelial remodeling and regenerative programs (Figure 4B; Figures S6D and S6E). Heatmap analysis confirmed marked transcriptional shifts, most pronounced in the 5k condition. This transition is characterized by coordinated downregulation of canonical intestinal identity programs, including Lgr5^+^ crypt-base stemness (*Lgr5*, *Olfm4*, and *Smoc2*), WNT-dependent self-renewal (*Axin2* and *Znrf3*), Notch-mediated fate specification (*Dll1*, *Dll4*, and *Tead2*), and Paneth cell-associated antimicrobial function (*Defa5*, *Defa21*, and *Defa22*), accompanied by simultaneous upregulation of injury-responsive and progenitor-associated programs including *Ly6a*, *Tcf7l2*, *Numb*, *Areg*, *Lats2*, *Muc3*, *Lyz2*, and *Reg3g*. This reciprocal suppression of epithelial identity in favor of inflammatory adaptation is consistent with the recently described revival stem cell state and fetal-like epithelial reversion observed during intestinal injury and inflammation (Ayyaz et al., 2019), suggesting that macrophage-derived inflammatory signals are sufficient to drive this transcriptional switch in a density-dependent manner.

### Protein-level validation of epithelial remodeling under TNFα exposure

To further validate epithelial remodeling at the protein level, we analyzed stem cell-, secretory lineage-, and stress-associated markers by western blot following TNFα exposure and macrophage co-cultures (Figures 4C and 4D). Lgr5 protein levels remained low and did not substantially differ across TNFα-treated conditions, consistent with transcriptomic downregulation of canonical crypt-base programs. Lyz expression remained broadly preserved despite transcriptional reduction of Paneth-associated genes, suggesting maintenance of residual secretory features during remodeling. Sox9 protein was consistently elevated following TNFα exposure across monoculture and co-culture conditions, supporting the emergence of a progenitor-associated epithelial state, while Bmi1 was reduced across TNFα-treated groups, consistent with the transcriptomic shift away from canonical stem cell identity toward an injury-associated remodeling state. Tff3 levels, instead, remained stable despite transcriptional remodeling of mucin-associated genes. No significant changes were detected for Wnt3A, β-catenin, Notch1, Yap/Taz, pJnk, or cleaved caspase-3, suggesting that TNFα-induced epithelial remodeling was more prominently reflected at the transcriptional than at the protein level for the analyzed signaling mediators.

### DSS-induced intestinal inflammation shares inflammatory and epithelial remodeling features with TNFα-exposed co-cultures

**Figure 5:**
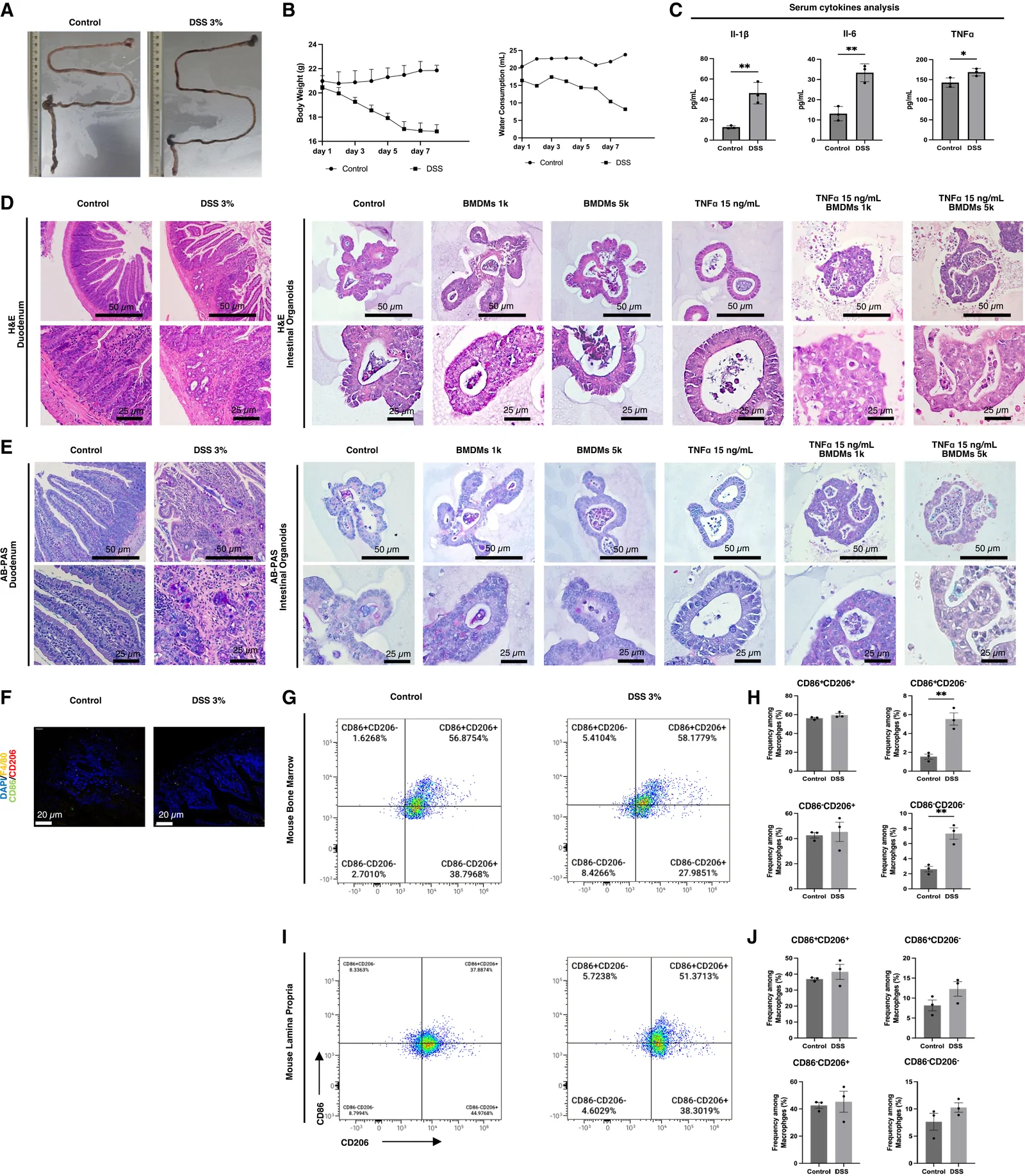
DSS-induced intestinal inflammation shares inflammatory and epithelial remodeling features with TNFα-exposed macrophage-organoid co-cultures (A) Macroscopic images of intestinal tissue from control and DSS-treated (3%) mice. (B) Body weight and water consumption monitored daily over 7 days (*n* = 5 mice/group). (C) Serum Il-1β, Il-6, and TNFα quantification in control and DSS-treated mice (*n* = 5 mice/group). (D) Representative H&E staining of duodenal sections from control and DSS-treated mice (left) and organoids across all experimental conditions (right). Scale bars: 50 μm (upper) and 25 μm (lower). (E) Representative Alcian blue-PAS staining of duodenal sections and organoids across conditions. Scale bars: 50 μm (upper) and 25 μm (lower). (F) Immunofluorescence of duodenal sections stained for DAPI, F4/80, CD86, and CD206. Scale bars: 20 μm. Representative images from 5 independent experiments. (G) Flow cytometry dot plots of CD86/CD206 distribution in bone marrow macrophages from control and DSS-treated mice. (H) Quantification of macrophage subpopulations from bone marrow (*n* = 5 mice/group). (I) Flow cytometry dot plots of CD86/CD206 distribution in lamina propria macrophages from control and DSS-treated mice. (J) Quantification of macrophage subpopulations from lamina propria (*n* = 5 mice/group). Data: mean ± SEM. Statistics: unpaired Student’s *t* test for two-group comparisons; one-way ANOVA with Tukey’s post hoc test for multiple comparisons. ^∗^*p* < 0.05, ^∗∗^*p* < 0.01, ^∗∗∗^*p* < 0.001, ^∗∗∗∗^*p <* 0.0001.

To evaluate the physiological relevance of the co-culture system, we established a DSS-induced intestinal inflammation model and compared selected inflammatory and epithelial remodeling features with TNFα-treated co-cultures (Figure 5). Mice receiving 3% DSS for 7 days displayed progressive body weight loss, reduced water consumption, and visually reduced intestinal length, consistent with established features of DSS-induced colitis (Figures 5A and 5B). Serum cytokine analysis revealed increased Il-1β, Il-6, and TNFα in DSS-treated animals, with Il-6 showing the most prominent increase, consistent with the cytokine profile observed in TNFα-treated co-cultures (Figure 5C). H&E staining of duodenal tissue demonstrated marked epithelial remodeling including altered villus organization, crypt distortion, and inflammatory tissue architecture in DSS-treated mice (Figure 5D). Similar morphological alterations were observed in TNFα-treated organoids, which displayed loss of budding structures and transition toward rounded morphologies, whereas untreated co-cultures maintained more preserved crypt-like architecture. TNFα-treated co-cultures additionally displayed macrophage infiltration within the organoid compartment, consistent with immune-epithelial interactions observed throughout the model characterization.

### DSS tissues and TNFα exposed co-cultures display comparable secretory and junctional remodeling features

To further assess epithelial secretory remodeling, Alcian blue-PAS staining was performed on both DSS intestinal tissues and organoid co-cultures (Figure 5E). Alcian blue-PAS staining demonstrated qualitatively increased acidic mucin staining in DSS-treated duodenal tissues compared with controls, with comparable enhanced Alcian blue-positive staining observed in TNFα-treated co-cultures relative to untreated organoids, indicating parallel secretory remodeling responses (Figure 5E). Immunofluorescence for Muc2 and Tff3 revealed increased Muc2 staining in both DSS-treated intestines and TNFα-treated co-cultures, whereas Tff3 remained stable across organoid conditions, suggesting selective remodeling of mucus-associated epithelial programs (Figure S7A).

Fluorescein isothiocyanate-dextran permeability assays demonstrated increased luminal fluorescence in co-culture conditions, most pronounced in the 1k BMDM condition, potentially reflecting stochastic epithelial disruption at low macrophage density, whereas higher macrophage numbers may promote a more coordinated epithelial response partially preserving junctional integrity (Figures S7B and S7C). E-cadherin immunofluorescence revealed increased signal intensity in both DSS-treated intestines and TNFα-treated co-cultures compared with respective controls, supporting epithelial junctional remodeling under inflammatory conditions (Figure S7D). These findings demonstrate that TNFα-treated macrophage-organoid co-cultures recapitulate selected secretory and junctional remodeling features observed in DSS-induced intestinal inflammation *in vivo*, supporting the physiological relevance of the platform for modeling epithelial inflammatory responses under controlled experimental conditions.

### Macrophage phenotypes in DSS tissues partially overlap with inflammatory co-culture states

To compare macrophage-associated phenotypes between the *in vitro* and *in vivo* inflammatory settings, macrophage populations were analyzed by immunofluorescence and flow cytometry using F4/80, CD86, and CD206 markers in both BMDM co-cultures and DSS-derived lamina propria immune cells (Figures 5F–5J; Figure S7E). DSS-derived BMDMs displayed increased proportions of CD86^+^ macrophages together with enrichment of double-negative populations compared with control conditions, indicating inflammatory-associated shifts in macrophage marker distribution following DSS exposure. In contrast, macrophages isolated from the intestinal lamina propria showed heterogeneous CD86/CD206 population distributions in DSS-treated animals, including a comparable trend toward CD86^+^ populations and CD86^−^/CD206^−^ double-positive populations, although these differences did not reach statistical significance. Together, these results indicate that both DSS-induced inflammation and TNFα-treated co-cultures are associated with heterogeneous macrophage phenotypic remodeling characterized by comparable shifts in CD86/CD206 marker distribution across *in vivo* and *in vitro* conditions.

## Discussion

In the present study, we established a macrophage-organoid co-culture platform that enables controlled investigation of epithelial-immune interactions under both homeostatic and inflammatory conditions. Recent studies have highlighted the importance of direct macrophage-epithelial communication in intestinal biology, including the work by Moraitis et al., (2025) demonstrating that mucosal macrophages govern intestinal epithelial regeneration following injury through NRG1 and SPP1 macrophage-derived signaling in a similar organoid-macrophage co-culture system. Consistent with these findings, our system captures macrophage-dependent modulation of epithelial organization and transcriptional programs, extending this framework to inflammatory conditions induced by TNFα stimulation. Several co-culture strategies combining intestinal organoids with primary macrophages, macrophage cell lines, or other immune cells have been recently described (Nozaki et al., 2016; Kakni et al., 2022; Hentschel et al., 2024), highlighting the growing interest in immune-competent organoid platforms. By combining murine intestinal organoids with BMDMs, this system maintained crypt-like epithelial organization while supporting direct immune-epithelial communication and inflammatory responsiveness. Importantly, macrophage density critically influenced epithelial stability within the co-culture system. While high macrophage concentrations induced spheroid-like epithelial morphologies and loss of crypt architecture, lower densities more effectively preserved epithelial organization. Among the tested conditions, the 5k BMDM co-culture provided the most consistent balance between epithelial preservation and inflammatory responsiveness. Under basal conditions, macrophage-containing co-cultures maintained stem cell localization, epithelial compartmentalization, and lineage-associated epithelial programs, while simultaneously displaying coordinated inflammatory-associated cytokine and transcriptional responses that were absent in organoid monocultures. Following TNFα exposure, the co-culture system underwent marked epithelial remodeling characterized by loss of canonical budding morphology, increased epithelial circularity, and transcriptional reprogramming of stem cell-associated pathways. Bulk RNA sequencing revealed reduced expression of canonical crypt-base stem cell markers together with enrichment of progenitor-, inflammatory-, and injury-associated transcriptional programs, consistent with a transition toward an inflammatory remodeling state rather than complete epithelial collapse. These changes were accompanied by increased Sox9 expression, mucus-associated remodeling, and junction-associated epithelial alterations, supporting the emergence of a coordinated epithelial stress response within the inflammatory co-culture environment. To further evaluate the physiological relevance of the system, we compared selected inflammatory and epithelial remodeling features with a DSS-induced intestinal inflammation model *in vivo*. DSS-treated tissues displayed increased inflammatory cytokine levels, altered epithelial organization, mucus-associated remodeling, and junctional alterations, several of which were similarly observed in TNFα-treated co-cultures. Although the present system does not recapitulate the full complexity of intestinal inflammatory disease, these overlapping features support its utility for studying selected aspects of inflammatory epithelial-immune remodeling within a controlled experimental setting.

Several limitations of the current model should be acknowledged. First, the co-culture system was intentionally designed as a short-term inflammatory platform to preserve epithelial organization and avoid progressive epithelial destabilization associated with prolonged immune co-culture. In addition, the present model contains only epithelial and macrophage compartments and, therefore, does not fully reproduce the multicellular complexity of the intestinal niche. The absence of stromal populations, including tissue-resident fibroblasts, as well as additional immune cell types such as T cells, dendritic cells, and innate lymphoid cells, represents an important limitation when compared with the *in vivo* intestinal microenvironment (Papp et al., 2024). Future incorporation of stromal populations, including tissue-resident fibroblasts, together with exposure to additional inflammatory cytokines, microbial metabolites, and pathogen-associated stimuli, may further expand the biological relevance of the system. Importantly, while bulk RNA sequencing enabled broader characterization of epithelial and inflammatory remodeling, higher-resolution approaches such as single-cell RNA sequencing will be valuable to further dissect epithelial and macrophage heterogeneity within the co-culture environment.

The exclusive use of duodenal organoids limits regional differences in epithelial composition, immune-cell distribution, microbial exposure, and inflammatory susceptibility, which are well recognized along the intestinal tract and are likely to influence epithelial-immune interactions under inflammatory conditions. To maintain anatomical consistency, the *in vivo* DSS comparisons were, therefore, performed using duodenal tissue corresponding to the origin of the organoid cultures (Hentschel et al., 2024; Moraitis et al., 2025). The present model should be interpreted as a small intestine-oriented inflammatory epithelial platform rather than as a comprehensive model of colonic inflammatory bowel disease pathology.

Overall, this study establishes a controlled immune-competent intestinal organoid platform that captures selected inflammatory and epithelial remodeling features associated with intestinal inflammatory stress while maintaining experimental accessibility and epithelial organization. Compared with existing organoid-macrophage co-culture approaches cited above, the present system extends current platforms by incorporating density-dependent macrophage effects, TNFα-driven inflammatory remodeling, and parallel *in vivo* validation in a DSS-induced intestinal inflammation model. Future studies leveraging this platform may contribute to the development of more physiologically relevant preclinical models for investigating intestinal inflammatory diseases, host-immune interactions, and potential therapeutic strategies targeting the epithelial-immune axis.

## Resource availability

### Lead contact

The data that support the findings of this study, which are already not provided, are available from the lead contact, Changhwan Ahn (cahn@jejunu.ac.kr).

### Materials availability

The authors confirm that all the relevant materials supporting the findings of this study are included in the paper/and or its supplemental information files.

### Data and code availability

All raw and processed data, together with their full annotated R code for all computational analyses, are deposited in public repositories upon publication (https://github.com/taebbio-JUN/BMDMs-co-culture-project---Stem-Cell-Reports/blob/main/README.md). Bulk RNA sequencing data are deposited in the ENA website (ENA: PRJEB113059,ERP193485). All datasets follow FAIR and CARE principles.

## Acknowledgments

This work was supported by the 10.13039/501100003725National Research Foundation of Korea grant funded by the Korea government (MSIT) (no. RS-2025-24535041).

## Author contributions

L.T. was responsible for the study conception, design, model construction, collection and assembly of the data, data analyses, and writing of the manuscript. T.B.L. was responsible for model construction, collection and assembly of the data, and bioinformatics data analyses. E.J. was responsible for model construction, collection and assembly of the data, and data analyses. S.H. was responsible for bioinformatics data analyses. S.C. was responsible for collection of data. C.A. was responsible for the study conception and design, data interpretation, revision of the manuscript, funding acquisition, and final approval of the manuscript.

## Declaration of interests

The authors declare no competing interests.

## Declaration of generative AI and AI-assisted technologies in the writing process

No AI and/or AI-assisted technologies were used in the writing process.

## STAR★Methods

### Key resources table

**Table:** 

REAGENT or RESOURCE	SOURCE	IDENTIFIER
**Antibodies**		

Anti-mouse	Cell Signaling	#7076; RRID: AB_330924
Anti-mouse Alexa 594	Invitrogen	#A21203; RRID: AB_141633
Anti-rabbit	Cell Signaling	#7074; RRID: AB_2099233
Anti-rabbit Alexa 488	Invitrogen	#A11008; RRID: AB_143165
Anti-rabbit Alexa 594	Invitrogen	#A11012; RRID: AB_2534079
Anti-rat	ThermoScientific	#31470; RRID: AB_228356
Anti-rat Alexa 488	Invitrogen	#A11006; RRID: AB_141373
APC anti-mouse CD206	BioLegend	#141708; RRID: AB_10900231
B-actin	Invitrogen	#15G5A11/E2; RRID: AB_2536844
B-catenin	Cell Signaling	#37447; RRID: AB_3246427
Bmi1	Abcam	#ab254253; RRID: AB_3073892
CD24	Invitrogen	#14-0242-82; RRID: AB_467170
Cleaved-caspase 3	Cell Signaling	#9664; RRID: AB_2070042
FITC anti-mouse CD86	Elabscience	#E-AB-F0994UC; RRID: AB_3665944
Ki67	Abcam	#16667; RRID: AB_302459
Lgr5	BD Pharmingen	#562731; RRID: AB_2737752
LIVE/DEAD™ Fixable Aqua Dead Cell Stain Kit	Invitrogen	#L34966
Lysozyme	Cell Signaling	#60487; RRID: AB_3741672
Muc2	Novusbio	#NB120-11197; RRID: AB_791261
Notch1	Cell Signaling	#3608; RRID: AB_2153354
PE anti-mouse CD326	Invitrogen	#12-5791-82; RRID: AB_1134176
PE anti-mouse F4/80	BioLegend	#123110; RRID: AB_893498
PerCP anti-mouse CD45	BioLegend	#103129; RRID: AB_893343
Phospho-SAPK/JNK	Cell Signaling	#9251; RRID: AB_331659
Sox9	Abcam	#ab185230; RRID: AB_2715497
Tff3	Invitrogen	#PA5-21081; RRID: AB_11152862
Wnt3a	Cell Signaling	#2721; RRID: AB_2215411
YAP/TAZ	Cell Signaling	#8418; RRID: AB_10950494

**Biological samples**		

Murine Bone Marrow derived Macrophages	This study	N/A
Murine Intestinal Organoids	This study	N/A
Murine Lamina propria Macrophages	This study	N/A

**Chemicals, peptides, and recombinant proteins**		

Accutase	Invitrogen	00-4555-56
ACK Lysing Buffer	Gibco	A1049201
Alcian blue	Solarbio	–
Ambion™ DNase	ThermoFisher	#90083
antibiotic–antimycotic solution	Welgene	LS203-01
Bio-Rad Clarity Western ECL Substrate	Bio-Rad	#1705061
BSA	Sigma	A5611-10G
Collagenase IV	STEMCELL Technologies	#07907
DMEM	Gibco	#11965092
DSS	MP Biomedicals	#160110
EDTA	Biosesang	#E2007
FBS	Gibco	#16000-044
FITC-dextran	Sigma	#42867-5G
HistoGel	Epredia	HG-4000-012
Immersol 518F	Zeiss	#433802-9010-000
IntestiCult Organoid Growth Medium	STEMCELL Technologies	#06005
L929 Conditioned medium	This study	N/A
Mayer’s hematoxylin	Dako	S3309
Penicillin/streptomycin	Gibco	#15140-122
Periodic Acid	Diapath	G0162
phenol red-free Matrigel	Corning	#356231
Primocin (1x)	InvivoGen	Ant-pm-1
Proteinase inhibitors	ThermoFisher	#A37989
Recombinant murine TNFα	ThermoFisher	#31501A20UG
RIPA buffer	ThermoFisher	#89900
RPMI 1640	Corning	#10-043-CVR
Schiff reagent	Diapath	C0612
Triton X-100	Sigma-Aldrich	#9036-19-5
TRIzol reagent	Invitrogen	#15596-026
TruStain FcX™ PLUS	BioLegend	#156603

**Critical commercial assays**		

M-CSF ELISA kit	Elabscience	#E-MSEL-M0105
Mouse IL-1β ELISA kit	Abcam	#197742
Mouse IL-6 ELISA kit	Abcam	#222503

**Deposited data**		

RNA-bulk seq data of mouse Intestinal Organoids, moo-cultures and co-cultures, and murine BMDM	This study	ENA: PRJEB113059, ERP193485

**Experimental models: Cell lines**		

L929 cell line	ATCC	NCTC clone 929

**Experimental models: Organisms/strains**		

C57BL/6J	SamTako	–

**Software and algorithms**		

FlowJo v10	BD Biosciences	https://flowjo.com
GraphPad Prism v9	GraphPad Software	https://www.graphpad.com
ImageJ	NIH	https://imagej.net/ij/

**Other**		

GM500 and DGM rack	Tecniplast	–

### Experimental model and study participant details

#### Mice

Male mice C57BL/6J aged 4–6 months were purchased from samtako (Osan Korea) and maintained in gm500 for mice and dgm rack (tecniplast). All mouse experiments were performed in accordance with the guidelines of Jeju National University approved by the institutional animal care and use committee (protocol number 2024-0014).

#### DSS model induction

DSS-induced intestinal inflammation was established by administering 3% DSS (#160110, MW 36,000–50,000) in drinking water for 7 days, with fresh solution replaced every other day. Control mice received regular drinking water. Body weight and water consumption were monitored daily. At endpoint, mice were euthanized by CO_2_ asphyxiation and blood collected by cardiac puncture. Serum was obtained by centrifugation at 2000 × g for 10 min at 4°C and stored at −80°C. Duodenal tissues were collected for histology, immunofluorescence, flow cytometry, and ELISA analyses.

#### Mouse intestinal organoids isolation and culture

Mouse duodenal tissues (∼15 cm distal to the pylorus) were harvested, washed in cold PBS, opened longitudinally, and cut into 2 mm fragments. Tissues were incubated in 2.5 mM EDTA (Biosesang, E2007) for 15 min at room temperature on an orbital shaker. After additional PBS washes, crypt fractions were collected through a 70 μm cell strainer, centrifuged at 300 × g for 3 min, resuspended in cold PBS, and counted using a hemocytometer. Approximately 300 crypts were embedded in 30 μL of growth factor-reduced, phenol red-free Matrigel (Corning, #356231) and plated in 48-well plates (Corning, #3548). Following Matrigel polymerization at 37°C for 10 min, 250 μL of IntestiCult Organoid Growth Medium (STEMCELL Technologies, #06005) supplemented with 1% penicillin/streptomycin (Gibco, #15140-122) was added per well. Medium was changed every other day. Organoid morphological parameters including budding number, circularity, organoid area, and diameter were quantified from brightfield images using Fiji (ImageJ). At least 40 organoids per condition were analyzed across 5 independent experiments.

#### L929 conditioned medium

L929 murine fibroblasts were maintained in DMEM supplemented with 10% FBS and 1% antibiotic–antimycotic solution at 37°C in 5% CO_2_. Only cells between passages 10 and 14 were used. At ∼90% confluence, cells were subcultured at a 1:5 ratio into T75 flasks containing 15 mL of complete medium with no subsequent medium addition or replacement. Conditioned medium was harvested at days 1, 3, 5, 7, 10, and 14 following phase-contrast imaging, centrifuged at 300 × g for 5 min to remove cellular debris, filtered through a 0.22 μm membrane, and stored at −20°C until use. M-CSF concentrations were quantified by ELISA (Elabscience #E-MSEL-M0105) across time points, and day-7 L929-conditioned medium showing optimal M-CSF levels was used for all downstream applications.

#### Bone marrow-derived macrophages culture

Bone marrow was isolated from femurs and tibiae of adult C57BL/6J mice under sterile conditions and flushed with ice-cold PBS supplemented with 1× Primocin (InvivoGen, ant-pm-1). The suspension was dissociated by repeated pipetting and centrifuged at 300 × g for 5 min. Erythrocytes were lysed in ACK Lysing Buffer (Gibco, A1049201) for 15 min at room temperature, and the suspension filtered through a 70-μm cell strainer. Cells were resuspended in differentiation medium consisting of DMEM supplemented with 10% FBS, 1% antibiotic–antimycotic solution (Welgene, LS203-01), 1× Primocin, and 20% L929-conditioned medium as M-CSF source. Three million cells per animal were seeded into 75-cm^2^ flasks and cultured at 37°C in 5% CO_2_ for 3 days to obtain BMDMs. All BMDM batches were tested for mycoplasma contamination prior to use. For harvesting, adherent BMDMs were incubated with Accutase (Invitrogen, 00-4555-56) for 15 min at 37°C; remaining adherent cells were detached with a cell scraper and complete detachment confirmed by phase-contrast microscopy.

### Method details

#### Intestinal organoids and BMDMs co-culture

At day 3 of organoid culture, the medium was aspirated and replaced with 250 μL of IntestiCult Organoid Growth Medium (STEMCELL Technologies #06005) containing either 1 × 10^3^ or 5 × 10^3^ BMDMs per well. BMDMs were added directly to the medium overlying the Matrigel rather than embedded within it, to preserve spontaneous macrophage transmigration toward the organoid compartment. Prior to co-culture, BMDMs had been differentiated for 3 days in DMEM supplemented with 10% FBS and 20% L929-conditioned medium as described above. Co-cultures were maintained at 37°C in 5% CO_2_ for 24 h. Where indicated, TNFα (15 ng/mL) was added to the co-culture medium simultaneously with BMDMs at the initiation of co-culture (Figure 1A).

#### TNFα exposure

Naive organoids and organoids–BMDM co-cultures were stimulated with recombinant murine TNFα (ThermoFisher #31501A20UG) at a final concentration of 15 ng/mL after 3 days of culture, once stable morphology and viability were established. TNFα was directly added to the culture medium without altering Matrigel composition, while control groups received vehicle only.

#### Cytokine quantification by ELISA

Supernatants from organoid monocultures and organoid–BMDM co-cultures were collected after 24 h at day 3 of co-culture. Mouse Il-1β (Abcam, #197742) and Il-6 (Abcam, #222503) were quantified by sandwich ELISA according to manufacturer’s instructions. Absorbance was measured at 450 nm with 570 nm reference correction using a BioTek microplate reader, and concentrations determined from standard curves (R^2^ > 0.99). The same procedure was applied to serum samples from DSS-treated mice.

#### Protein isolation and western blotting

Organoids were collected in 2.5 mM EDTA, centrifuged, and lysed in RIPA buffer supplemented with 1% protease inhibitor cocktail. Protein concentrations were determined using the Pierce BCA Protein Assay Kit (ThermoFisher). Equal amounts of protein (15 μg/30 μL) were separated by SDS-PAGE on 10% gels and transferred onto 0.45 μm nitrocellulose membranes. Membranes were blocked with 5% BSA in TBS-T for 1h at room temperature, incubated with primary antibodies overnight at 4°C, and with HRP-conjugated secondary antibodies for 1h at room temperature. Signals were detected using Bio-Rad Clarity Western ECL Substrate and quantified using Fiji (ImageJ). Primary and secondary antibodies with catalog numbers and dilutions are listed in Table S1.

#### Immunofluorescence in intestinal organoids and duodenum

Intestinal organoids were fixed in 4% PFA for 30 min and duodenal tissues for 24 h at room temperature. Paraffin-embedded tissue sections were deparaffinized, rehydrated, and subjected to heat-induced antigen retrieval in citrate buffer (10 mM, pH 6.0) at 95°C for 20 min prior to permeabilization. All samples were permeabilized in DPBS containing 5% horse serum and 0.5% Triton X-100 overnight at 4°C. Primary antibodies diluted in permeabilization buffer were incubated for 2 h at room temperature followed by overnight at 4°C. After DPBS washes, fluorophore-conjugated secondary antibodies (Invitrogen) were applied for 2 h at room temperature. Nuclei were counterstained with DAPI (5 μg/mL, 30 min). Images were acquired on a BC43 CF Confocal Microscope (Oxford Instruments). Primary and secondary antibodies with catalog numbers and dilutions are listed in Table S2.

#### Epithelial barrier permeability assay with FITC-dextran 40kDa

Epithelial barrier function was assessed using FITC-dextran (40 kDa, Sigma, #42867-5G) diluted in IntestiCult (2 mg/mL) and added to cultures for 30 min at 37°C after 4 days of treatment. Organoids were washed with DPBS and imaged on a Leica DMi8 microscope with a GFP 488 filter. Luminal fluorescence was quantified using Fiji by measuring integrated density of manually defined ROIs around the organoid lumen, normalized to organoid area. At least 10 organoids per condition were analyzed.

#### Flow cytometry and gating strategy

BMDMs and organoids co-cultures were detached with ACCUTASE (STEMCELL Technologies #07922) at 37°C for 15 min 1 × 10^6^ cells per sample were resuspended in FACS buffer (PBS, 2 mM EDTA, 0.5% FBS) and Fc receptors blocked with TruStain FcX PLUS (BioLegend #156603) for 10 min at room temperature. Surface markers were stained using antibodies listed in Table S3, at 1:100 dilution, together with LIVE/DEAD Fixable Aqua (Invitrogen, #L34966) at 1:1000, for 30 min at 4°C in the dark. A minimum of 10,000 events within the macrophage gate were acquired per sample on a CytoFLEX LX (Beckman Coulter). Multicolor compensation was performed using single-stained controls. Viable single cells were gated sequentially on FSC/SSC, singlet discrimination, LIVE/DEAD negativity, CD45^+^, and F4/80^+^ to identify macrophages. CD86 and CD206 were analyzed within the CD45^+^F4/80^+^ gate, with gates established on unstimulated controls. For co-culture experiments, CD45 and EpCAM were used to distinguish immune and epithelial populations within the live single cell gate. Data analysis was performed using FlowJo v10 (BD Biosciences).

#### RNA isolation

Total RNA was isolated from intestinal organoids, BMDMs, and co-culture samples using TRIzol reagent (Invitrogen, #15596-026) according to the manufacturer’s instructions. Briefly, samples were homogenized in TRIzol reagent and phase separation was achieved by addition of chloroform (OCl company Ltd., #67-66-3) at a 1:5 (v/v) ratio, followed by vortexing and centrifugation at 12,000 × g for 15 min at 4°C. The upper aqueous phase was carefully collected and transferred to a fresh tube. RNA was precipitated by addition of an equal volume of isopropanol (Chembiotec, #5035-4100) and incubation overnight at −20°C. Precipitated RNA was collected by centrifugation at 18,000 rpm for 10 min at 4°C until a visible pellet formed. The supernatant was carefully discarded, and the RNA pellet was washed twice with 75% ethanol followed by centrifugation at 7,500 × g for 5 min at 4°C. The ethanol was removed and the pellet air-dried briefly before resuspension in an appropriate volume of DEPC-treated water (Invitrogen, #46–2224). RNA concentration and purity were assessed spectrophotometrically by measuring absorbance at 260 nm and 280 nm using a NanoDrop spectrophotometer; samples with A260/A280 ratio between 1.8 and 2.1 were considered acceptable for downstream applications. Reverse transcription to cDNA for quality validation purposes was performed using M-MLV reverse transcriptase (Invitrogen, #28025-013) according to the manufacturer’s instructions.

#### Bulk RNA sequencing: Library preparation and sequencing

Total RNA samples were submitted to Macrogen Inc. (Seoul, Republic of Korea) for library preparation, quality control, and sequencing. Strand-specific RNA sequencing libraries were constructed using the TruSeq Stranded Total RNA Library Prep Kit with Ribo-Zero Gold rRNA depletion (Human/Mouse/Rat; Illumina, San Diego, CA, USA), enabling comprehensive transcriptome profiling with removal of ribosomal RNA from mouse samples. Library quality control was performed at two levels: (1) library fragment size distribution was assessed using an Agilent 4200 TapeStation system with D1000 ScreenTape (Agilent Technologies, Santa Clara, CA, USA), with observed fragment sizes ranging from 281 to 315 bp across all 24 libraries, consistent with the expected range of 260–320 bp for TruSeq Stranded Total RNA libraries following adapter ligation and PCR enrichment; (2) library molar concentration was determined by qPCR following the Illumina Sequencing Library qPCR Quantification Protocol Guide, with all 24 libraries meeting Macrogen’s pass criterion of ≥10 nM (observed range: 74.0–467.7 nM). Library mass concentration was additionally assessed by fluorometric assay, with observed concentrations ranging from 14.4 to 95.0 ng/μL. All libraries passed quality control criteria prior to sequencing. Paired-end sequencing (2 × 150 bp) was performed on the Illumina NovaSeq X Plus platform (Illumina, San Diego, CA, USA).

#### Lamina propria macrophages isolation

Duodenal tissues were opened longitudinally, washed with cold PBS, and cut into 1 cm fragments. Epithelial cells were removed by two rounds of incubation in HBSS containing 5 mM EDTA and 1 mM DTT at 37°C for 20 min under agitation, followed by extensive washing with cold PBS. The remaining tissue was enzymatically digested in RPMI containing Collagenase IV (1 mg/mL; STEMCELL Technologies #07907) and Ambion DNase I (0.1 mg/mL; ThermoFisher #90083) at 37°C for 30 min with gentle shaking. Cell suspensions were filtered through a 70 μm cell strainer, centrifuged at 400 × g for 5 min, and resuspended in FACS buffer for downstream staining. Lamina propria macrophages were identified by flow cytometry using the gating strategy described above.

#### Histological processing and staining tissue and intestinal organoid

Tissues were fixed in 4% PFA for 24 h and organoids for 2 h, washed in PBS, and processed within 24 h. Fixed organoids were embedded in HistoGel (Epredia) within paraffin molds, solidified at 4°C, and processed alongside tissue specimens. Paraffin blocks were sectioned at 2 μm, deparaffinized in xylene, and rehydrated through graded ethanol. For H&E staining, sections were stained with Mayer’s hematoxylin (Dako, S3309) for 1 min, rinsed in tap water, and counterstained with Eosin Y (Sigma-Aldrich, 318906) for 15 min. For Alcian Blue–PAS staining, sections were incubated in Alcian blue (Solarbio) for 30 min, oxidized in Periodic Acid (Diapath, G0162) for 10 min, and treated with Schiff reagent (Diapath, C0612) for 30 min. All sections were dehydrated and mounted with Immersol 518F (#433802-9010-000). Images were acquired on a ZEISS Axio imager A2.

### Quantification and statistical analysis

#### Bulk RNA-sequencing data analysis

All analyses were performed in R v4.3.2. Gene-level count matrices produced upstream by HISAT2 alignment to the mouse reference genome (GRCm39/mm39) and StringTie transcript assembly with gene-level counting were imported into R, with composite identifiers of the form ENSEMBL.version|SYMBOL or MSTRG.X|SYMBOL parsed to gene-symbol level and annotated against org.Mm.e.g.,.db (v3.18.0) to recover SYMBOL, ENTREZID, and GENENAME fields; genes with ≥10 counts in ≥3 samples were retained for downstream analysis; library-level quality control comprised per-sample log_2_-count histograms and boxplots before and after filtering, detected-gene counts per sample, DESeq2-normalized count boxplots, principal component analysis on variance-stabilizing-transformed (VST) counts with vst(blind = FALSE), a sample-to-sample Euclidean distance heatmap on VST data, and per-gene dispersion estimates via plotDispEsts(); differential expression was performed in DESeq2 v1.42.0 using a one-factor design (∼group) encoding the eight biological conditions with median-of-ratios size factors and parametric dispersion estimation, and per-contrast effect sizes were stabilized using the adaptive shrinkage estimator implemented in ashr, with differentially expressed genes (DEGs) defined as those satisfying Benjamini–Hochberg adjusted *p* < 0.05 (Benjamini and Hochberg, 1995) and |log_2_ fold-change (LFC)| > 1, and transcript-level results collapsed to the most significant entry per ENTREZ ID; fourteen pairwise contrasts were organized into a six-tier hierarchy—Tiers 1 and 3 (five contrasts) using untreated organoid as the control to capture BMDM addition and cumulative TNF-α/TNF-α + BMDM effects on organoids, Tier 4 (two contrasts) using Org+TNFα to isolate the BMDM paracrine modifier on TNF-α–conditioned organoids, Tiers 2 and 5 (five contrasts) mirroring Tiers 1 and 3 from the macrophage perspective with untreated macrophage as the control, and Tier 6 (two contrasts) using Mac+TNFα to isolate the organoid paracrine modifier on TNF-α–conditioned macrophages—and reorganized into a four-baseline framework (Baseline 1 vs. untreated Org [Tiers 1 + 3]; Baseline 2 vs. Org+TNFα [Tier 4]; Baseline 3 vs. untreated Mac [Tiers 2 + 5]; Baseline 4 vs. Mac+TNFα [Tier 6]) to disentangle basal co-culture effects from TNF-α–conditioned paracrine modifiers; for two reference contrasts (Org+TNFα+BMDM 5K vs. Org+TNFα and Org+TNFα+BMDM 5K vs. Mac+TNFα), DESeq2 results were cross-validated by limma–voom (TMM-normalized DGEList with filterByExpr, voom precision-weighted modeling, lmFit + eBayes) and edgeR using glmQLFit + glmTreat at an LFC threshold of 1, with three-way DEG overlap (DESeq2 ∩ limma ∩ edgeR) tabulated, and pathway-level cross-validation provided by gprofiler2 (gost() with g_SCS multiple-testing correction) across GO:BP/MF/CC, KEGG, Reactome, and WikiPathways; functional enrichment was performed per contrast using clusterProfiler v4.10.1 on the DESeqDataSet-detected ENTREZ universe, with Gene Ontology over-representation analysis (ORA) conducted by enrichGO() (org.Mm.e.g.,.db) independently for the Biological Process, Molecular Function, and Cellular Component ontologies at *p*-value cutoff = 0.05 and q-value cutoff = 0.10, with redundant GO terms removed using clusterProfilersimplify() (cutoff = 0.7, by adjusted P), and KEGG pathway ORA performed by enrichKEGG() (organism = mmu) at *p*-value cutoff = 0.1 and q-value cutoff = 0.25 with setReadable() applied, alongside KEGG gene set enrichment analysis via gseKEGG() on ranked LFC vectors (*p*-value cutoff = 0.25, minGSSize = 10, maxGSSize = 500, fixed seed) and pathview-based LFC overlay on canonical KEGG pathway diagrams for the top five enriched pathways of each reference contrast; a weighted gene co-expression network was constructed in WGCNA (v1.72) on VST-transformed counts (vst(blind = TRUE)) from a parallel DESeqDataSet with design = ∼1, the soft-threshold power was selected as the lowest power in 1–20 yielding a signed scale-free topology fit R^2^ ≥ 0.80 (fallback = 14), module detection used blockwiseModules() (TOMType = “signed”, networkType = “signed”, maxBlockSize = 15,000, minModuleSize = 30, mergeCutHeight = 0.25, pamRespectsDendro = FALSE) under a fixed random seed, and module–trait correlations were computed against an 8-level group design matrix by Pearson correlation with Student-asymptotic P-values from corPvalueStudent(); per-contrast volcano plots were generated with EnhancedVolcano across all 14 contrasts, gene-level LFC matrices were rendered as unclustered heatmaps with pheatmap preserving gene and baseline-grouped contrast order, KEGG and GO enrichment results were visualized as four-baseline × 14-contrast bubble matrices via ggh4xfacet_grid2() with free x-scales by baseline (bubble size encoding gene count and bubble intensity encoding −log_10_(P)), and normalized counts were exported in Broad GSEA .gct/.cls format to support external workflows; core R packages were DESeq2 v1.42.0, ashr v2.2-63, apeglm, limma, edgeR, clusterProfiler v4.10.1, enrichplot v1.22.0, DOSE v3.28.2, pathview, gprofiler2, org.Mm.e.g.,.db v3.18.0, AnnotationDbi v1.64.1, EnhancedVolcano, WGCNA v1.72, ggplot2 v3.5.0, ggh4x v0.2.8, ggrepel v0.9.5, pheatmap v1.0.12, and the tidyverse, and all analysis code is available from the corresponding author upon reasonable request.

#### Statistical data analysis

Statistical analyses were performed using GraphPad Prism v9 (GraphPad Software, San Diego, CA). Comparisons between two groups were assessed by unpaired two-tailed Student’s *t* test. Multiple group comparisons were performed by one-way or two-way ANOVA followed by Tukey’s post-hoc test. Data are presented as mean ± SEM. A *p*-value <0.05 was considered statistically significant.

## Supplemental information

Document S1. Figures S1–S7 and Tables S1–S3

Document S2. Article plus supplemental information
