# Supplementary material for: A macrophage-intestinal organoid co-culture model captures balanced epithelial homeostasis and inflammation
Source: Stem Cell Reports. 2026 Aug 6;21(9):103045. doi: 10.1016/j.stemcr.2026.103045 (PMC13555428; doi:10.1016/j.stemcr.2026.103045)
Supplement: Supplementary file 1 [file mmc1.pdf]

Stem Cell Reports, Volume *21*

Supplemental Information

A macrophage-intestinal organoid co-culture model captures balanced epithelial homeostasis and inflammation

Lisa Tonini, Tae Baek Lee, Eunju Jang, Sungmoo Hong, Soobin Choi, and Changhwan Ahn

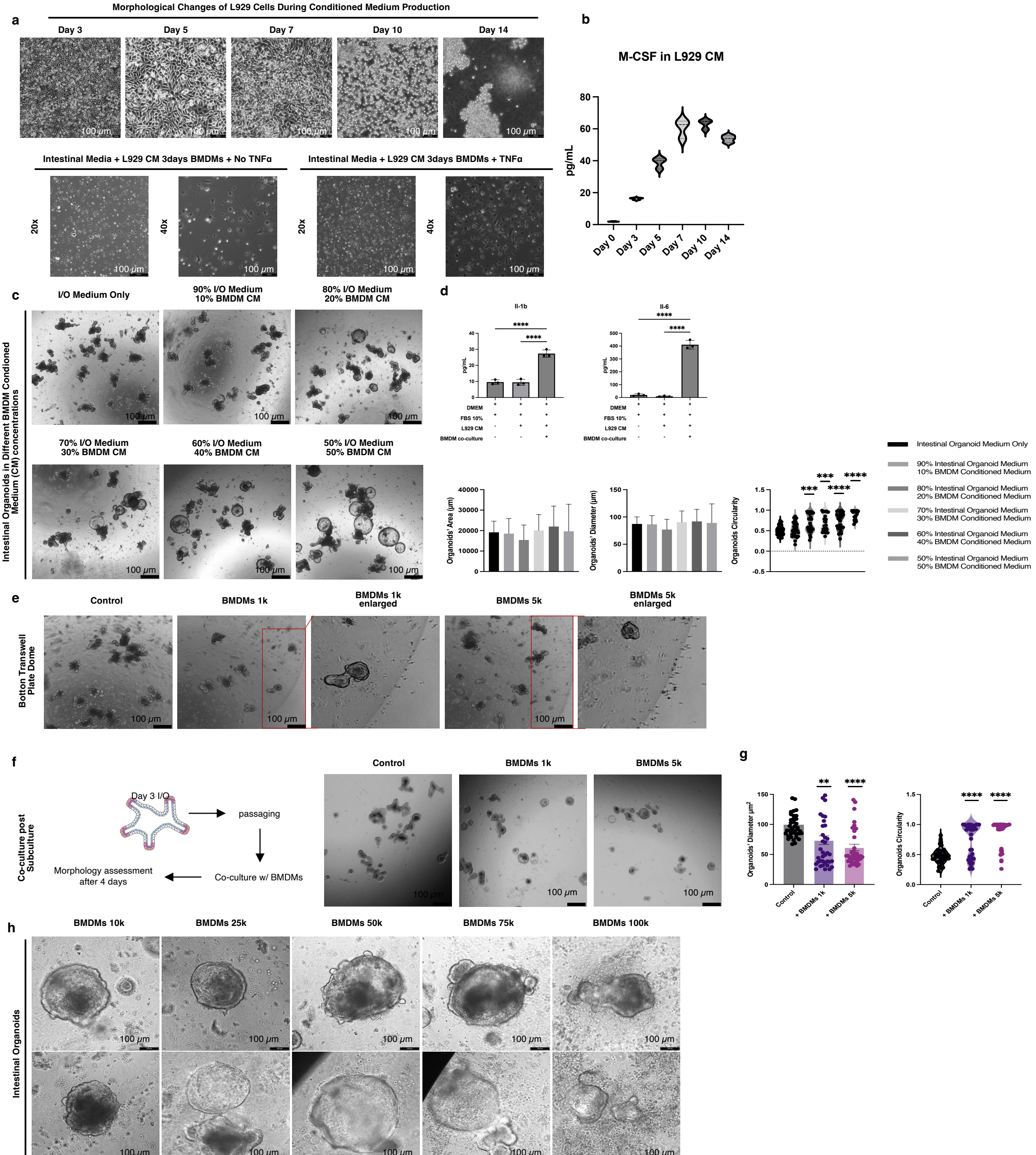

a
Morphological Changes of L929 Cells During Conditioned Medium Production
Day 3
Day 5
Day 7
Day 10
Day 14
100 µm
Intestinal Media + L929 CM 3days BMDMs + No TNFα
Intestinal Media + L929 CM 3days BMDMs + TNFα
20x
40x
b
M-CSF in L929 CM
pg/mL
Day 0
Day 3
Day 5
Day 7
Day 10
Day 14
c
I/O Medium Only
90% I/O Medium 10% BMDM CM
80% I/O Medium 20% BMDM CM
70% I/O Medium 30% BMDM CM
60% I/O Medium 40% BMDM CM
50% I/O Medium 50% BMDM CM
Intestinal Organoids in Different BMDM Condioned Medium (CM) concentrations
d
Il-1b
Il-6
DMEM
FBS 10%
L929 CM
BMDM co-culture
Intestinal Organoid Medium Only
90% Intestinal Organoid Medium 10% BMDM Conditioned Medium
80% Intestinal Organoid Medium 20% BMDM Conditioned Medium
70% Intestinal Organoid Medium 30% BMDM Conditioned Medium
60% Intestinal Organoid Medium 40% BMDM Conditioned Medium
50% Intestinal Organoid Medium 50% BMDM Conditioned Medium
Organoids' Area (µm)
Organoids' Diameter (µm)
Organoids Circularity
e
Control
BMDMs 1k
BMDMs 1k enlarged
BMDMs 5k
BMDMs 5k enlarged
Botton Transwell Plate Dome
f
Co-culture post Subculture
Day 3 I/O
passaging
Co-culture w/ BMDMs
Morphology assessment after 4 days
Control
BMDMs 1k
BMDMs 5k
g
Organoids' Diameter µm²
Organoids Circularity
Control
+ BMDMs 1k
+ BMDMs 5k
h
BMDMs 10k
BMDMs 25k
BMDMs 50k
BMDMs 75k
BMDMs 100k
Intestinal Organoids

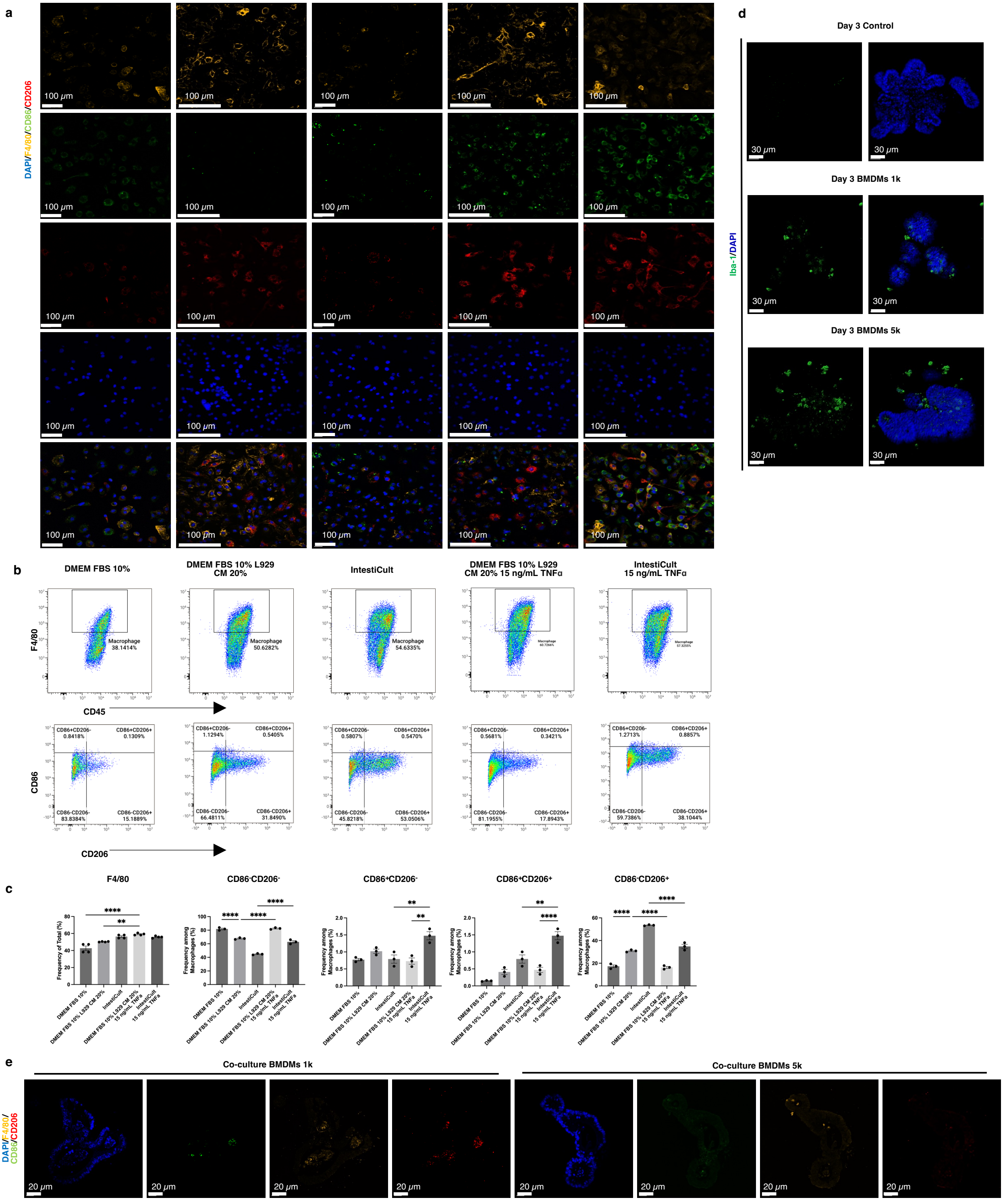

a
DAPI/F4/80/CD86/CD206
100 μm
b
DMEM FBS 10%
DMEM FBS 10% L929 CM 20%
IntestiCult
DMEM FBS 10% L929 CM 20% 15 ng/mL TNFα
IntestiCult 15 ng/mL TNFα
F4/80
CD45
Macrophage 38.1414%
Macrophage 50.6282%
Macrophage 54.6335%
CD86
CD206
CD86+CD206- 0.8418%
CD86+CD206+ 0.1309%
CD86-CD206- 83.8384%
CD86-CD206+ 15.1889%
CD86+CD206- 1.1294%
CD86+CD206+ 0.5405%
CD86-CD206- 66.4811%
CD86-CD206+ 31.8490%
CD86+CD206- 0.5807%
CD86+CD206+ 0.5470%
CD86-CD206- 45.8218%
CD86-CD206+ 53.0506%
CD86+CD206- 0.5681%
CD86+CD206+ 0.3421%
CD86-CD206- 81.1955%
CD86-CD206+ 17.8943%
CD86+CD206- 1.2713%
CD86+CD206+ 0.8857%
CD86-CD206- 59.7386%
CD86-CD206+ 38.1044%
c
F4/80
CD86-CD206-
CD86+CD206-
CD86+CD206+
CD86-CD206+
Frequency of Total (%)
Frequency among Macrophages (%)
DMEM FBS 10%
DMEM FBS 10% L929 CM 20%
IntestiCult
DMEM FBS 10% L929 CM 20% 15 ng/mL TNFa
IntestiCult 15 ng/mL TNFa
d
Day 3 Control
Day 3 BMDMs 1k
Day 3 BMDMs 5k
Iba-1/DAPI
30 μm
e
Co-culture BMDMs 1k
Co-culture BMDMs 5k
DAPI/F4/80/CD86/CD206
20 μm

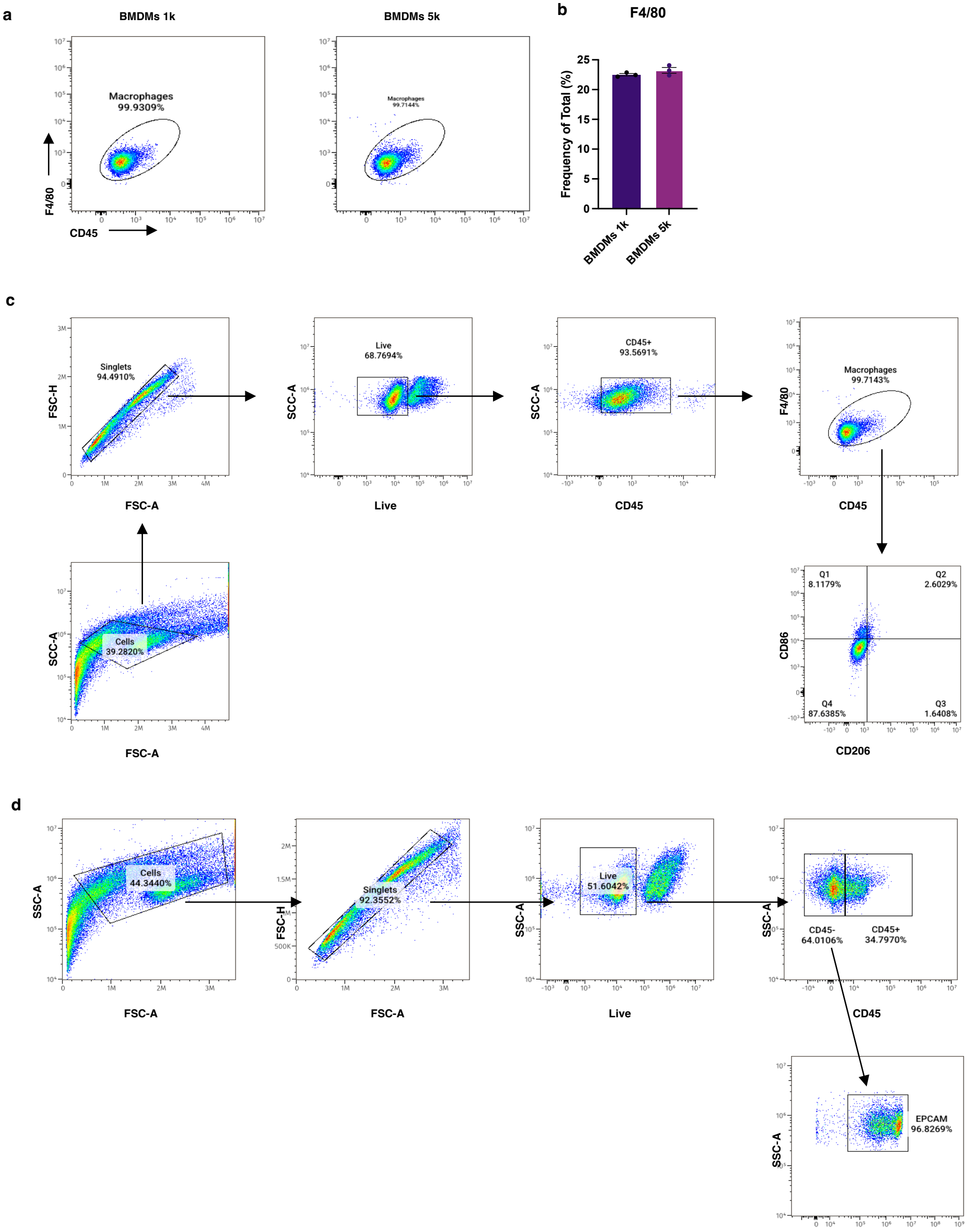

a
BMDMs 1k
Macrophages
99.9309%
F4/80
CD45
BMDMs 5k
Macrophages
99.7144%
b
F4/80
Frequency of Total (%)
25
20
15
10
5
0
BMDMs 1k
BMDMs 5k
c
Singlets
94.4910%
FSC-H
FSC-A
Live
68.7694%
SCC-A
Live
CD45+
93.5691%
SCC-A
CD45
Macrophages
99.7143%
F4/80
CD45
Cells
39.2820%
SCC-A
FSC-A
Q1
8.1179%
Q2
2.6029%
Q4
87.6385%
Q3
1.6408%
CD86
CD206
d
Cells
44.3440%
SSC-A
FSC-A
Singlets
92.3552%
FSC-H
FSC-A
Live
51.6042%
SSC-A
Live
CD45-
64.0106%
CD45+
34.7970%
SSC-A
CD45
EPCAM
96.8269%
SSC-A
EpCam

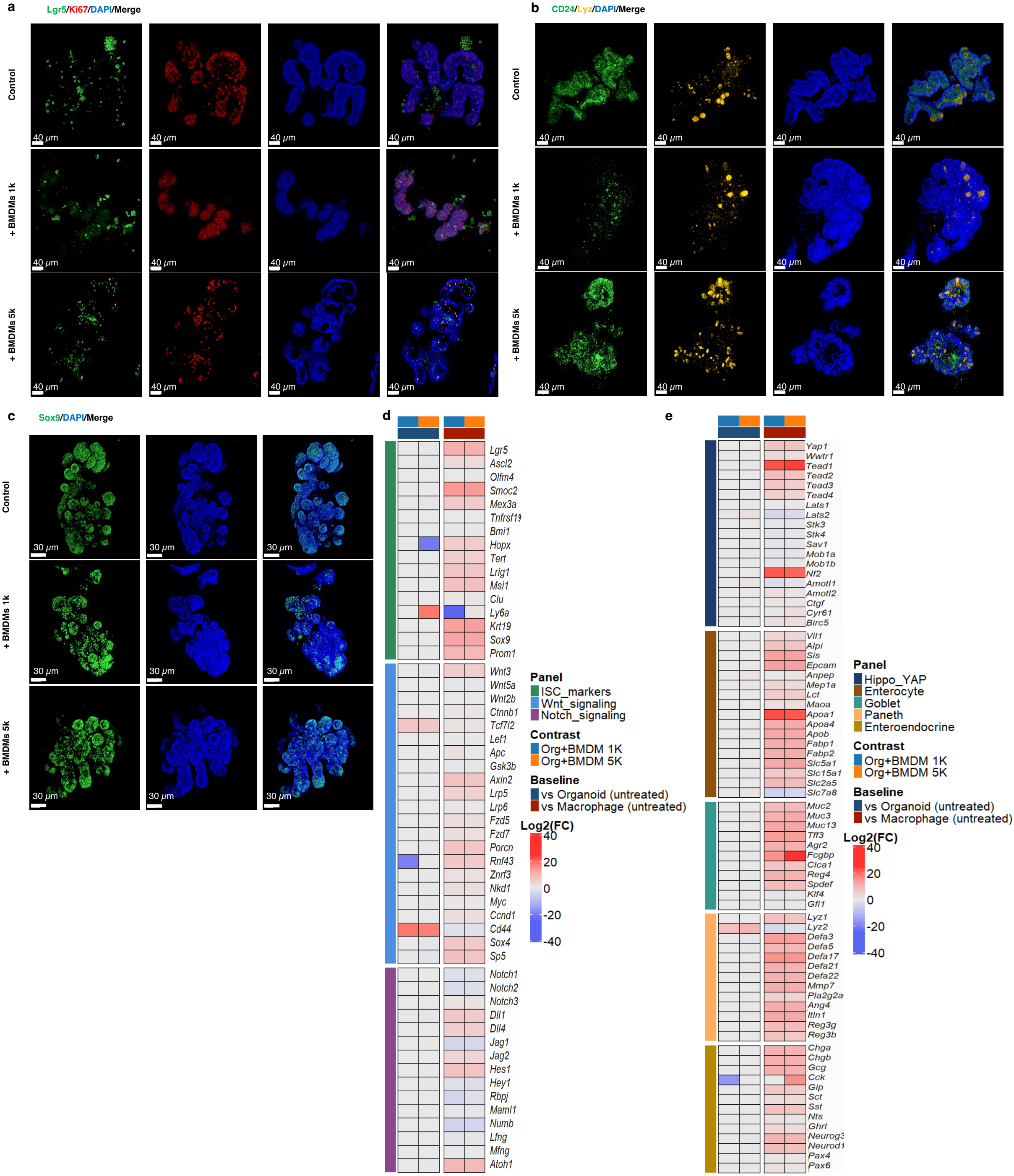
a
Lgr5/Ki67/DAPI/Merge
Control
+ BMDMs 1k
+ BMDMs 5k
40 μm
b
CD24/Lyz/DAPI/Merge
Control
+ BMDMs 1k
+ BMDMs 5k
40 μm
c
Sox9/DAPI/Merge
Control
+ BMDMs 1k
+ BMDMs 5k
30 μm
d
Lgr5
Ascl2
Olfm4
Smoc2
Mex3a
Tnfrsf19
Bmi1
Hopx
Tert
Lrig1
Msi1
Clu
Ly6a
Krt19
Sox9
Prom1
Wnt3
Wnt5a
Wnt2b
Ctnnb1
Tcf7l2
Lef1
Apc
Gsk3b
Axin2
Lrp5
Lrp6
Fzd5
Fzd7
Porcn
Rnf43
Znrf3
Nkd1
Myc
Ccnd1
Cd44
Sox4
Sp5
Notch1
Notch2
Notch3
Dll1
Dll4
Jag1
Jag2
Hes1
Hey1
Rbpj
Maml1
Numb
Lfng
Mfng
Atoh1
Panel
ISC_markers
Wnt_signaling
Notch_signaling
Contrast
Org+BMDM 1K
Org+BMDM 5K
Baseline
vs Organoid (untreated)
vs Macrophage (untreated)
Log2(FC)
40
20
0
-20
-40
e
Yap1
Wwtr1
Tead1
Tead2
Tead3
Tead4
Lats1
Lats2
Stk3
Stk4
Sav1
Mob1a
Mob1b
Nf2
Amotl1
Amotl2
Ctgf
Cyr61
Birc5
Vil1
Alpi
Sis
Epcam
Anpep
Mep1a
Lct
Maoa
Apoa1
Apoa4
Apob
Fabp1
Fabp2
Slc5a1
Slc15a1
Slc2a5
Slc7a8
Muc2
Muc3
Muc13
Tff3
Agr2
Fcgbp
Clca1
Reg4
Spdef
Klf4
Gfi1
Lyz1
Lyz2
Defa3
Defa5
Defa17
Defa21
Defa22
Mmp7
Pla2g2a
Ang4
Itln1
Reg3g
Reg3b
Chga
Chgb
Gcg
Cck
Gip
Sct
Sst
Nts
Ghrl
Neurog3
Neurod1
Pax4
Pax6
Panel
Hippo_YAP
Enterocyte
Goblet
Paneth
Enteroendocrine
Contrast
Org+BMDM 1K
Org+BMDM 5K
Baseline
vs Organoid (untreated)
vs Macrophage (untreated)
Log2(FC)
40
20
0
-20
-40

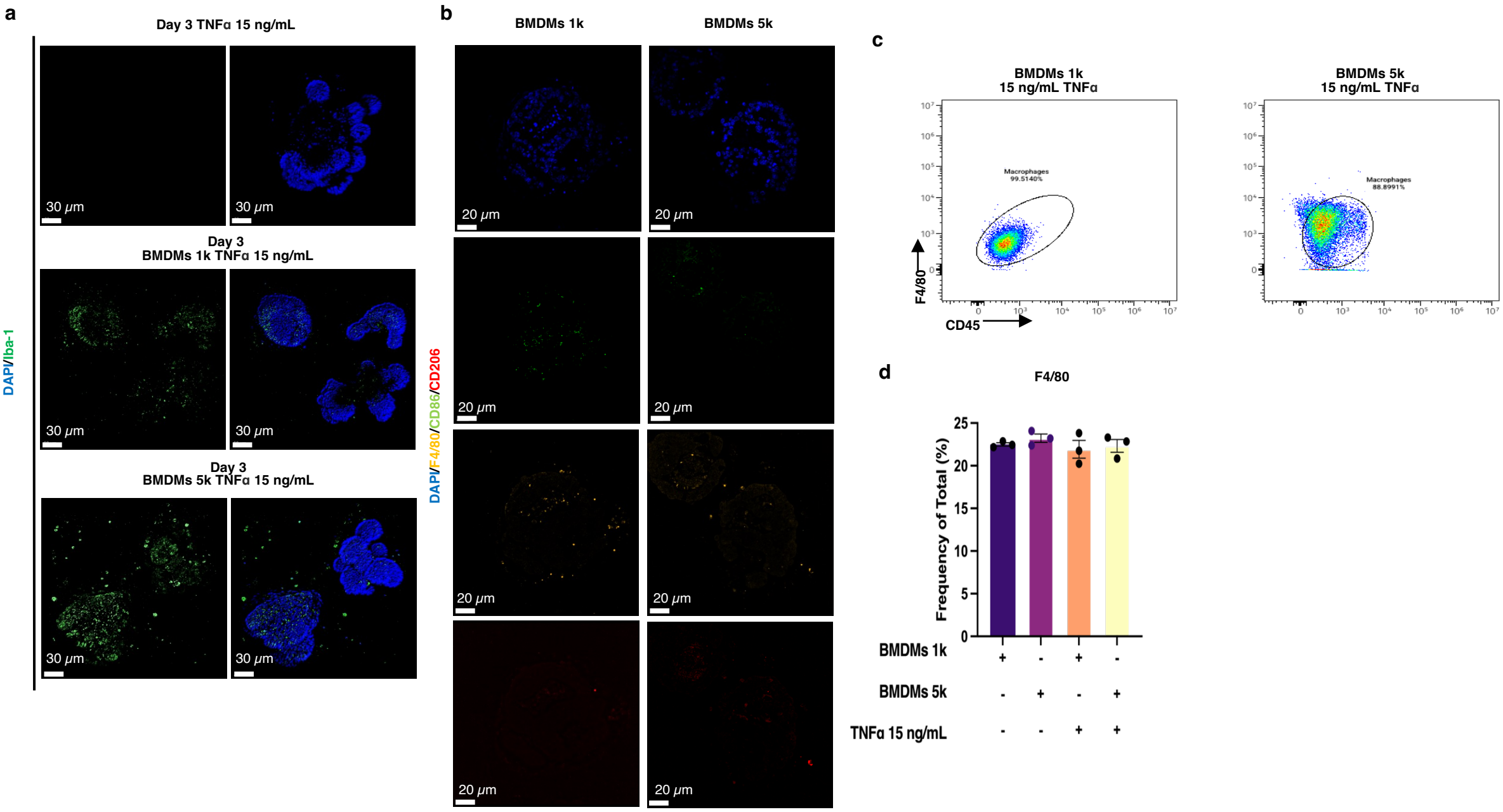
a
Day 3 TNFα 15 ng/mL
Day 3
BMDMs 1k TNFα 15 ng/mL
Day 3
BMDMs 5k TNFα 15 ng/mL
DAPI/Iba-1
30 μm
b
BMDMs 1k
BMDMs 5k
DAPI/F4/80/CD86/CD206
20 μm
c
BMDMs 1k
15 ng/mL TNFα
BMDMs 5k
15 ng/mL TNFα
Macrophages
99.5140%
Macrophages
88.8991%
F4/80
CD45
d
F4/80
Frequency of Total (%)
BMDMs 1k
BMDMs 5k
TNFα 15 ng/mL

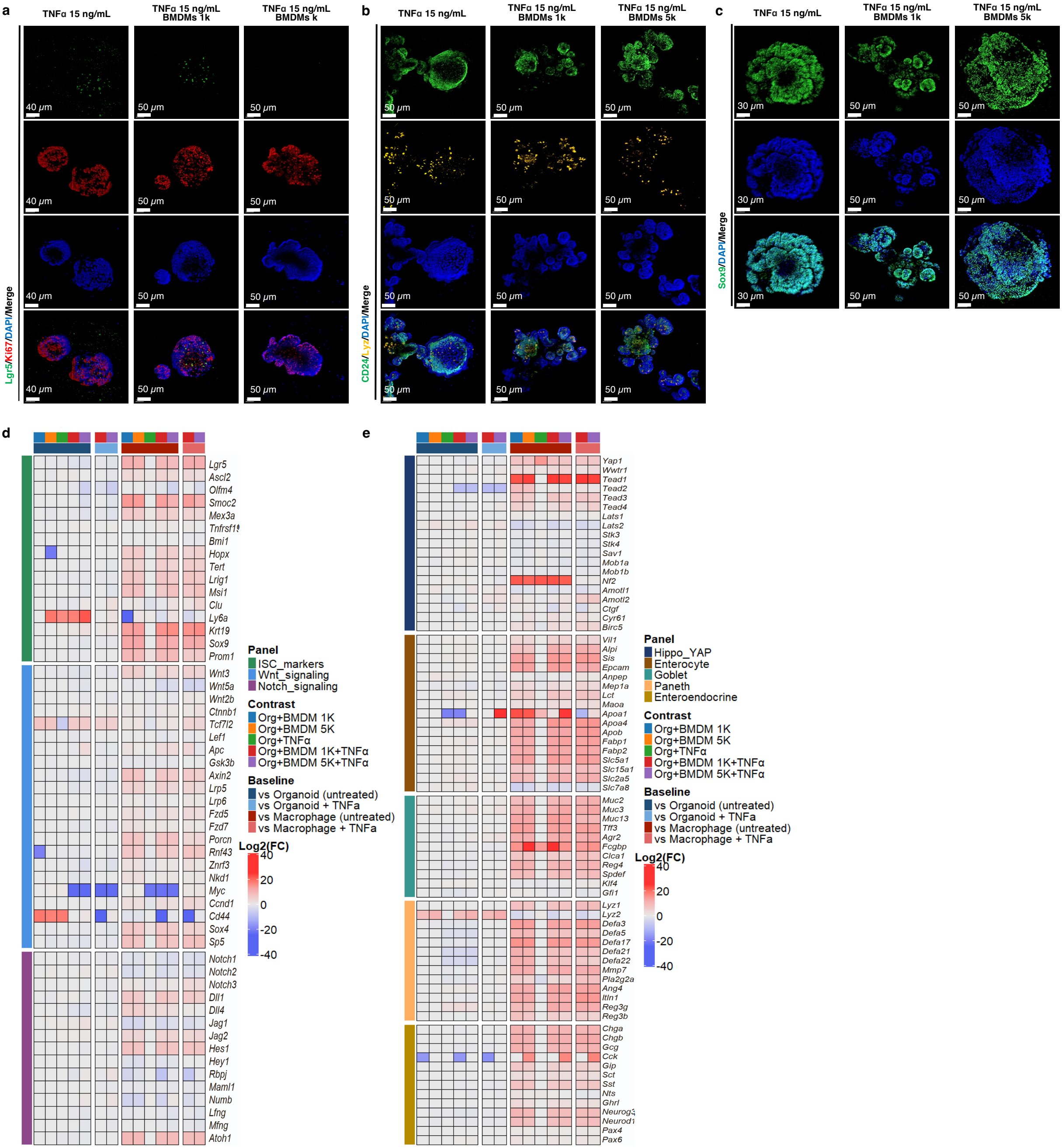

a
TNFα 15 ng/mL
TNFα 15 ng/mL BMDMs 1k
TNFα 15 ng/mL BMDMs k
Lgr5/Ki67/DAPI/Merge
40 μm
50 μm
b
TNFα 15 ng/mL
TNFα 15 ng/mL BMDMs 1k
TNFα 15 ng/mL BMDMs 5k
CD24/Lyz/DAPI/Merge
50 μm
c
TNFα 15 ng/mL
TNFα 15 ng/mL BMDMs 1k
TNFα 15 ng/mL BMDMs 5k
Sox9/DAPI/Merge
30 μm
50 μm
d
Lgr5
Ascl2
Olfm4
Smoc2
Mex3a
Tnfrsf19
Bmi1
Hopx
Tert
Lrig1
Msi1
Clu
Ly6a
Krt19
Sox9
Prom1
Wnt3
Wnt5a
Wnt2b
Ctnnb1
Tcf7l2
Lef1
Apc
Gsk3b
Axin2
Lrp5
Lrp6
Fzd5
Fzd7
Porcn
Rnf43
Znrf3
Nkd1
Myc
Ccnd1
Cd44
Sox4
Sp5
Notch1
Notch2
Notch3
Dll1
Dll4
Jag1
Jag2
Hes1
Hey1
Rbpj
Maml1
Numb
Lfng
Mfng
Atoh1
Panel
ISC_markers
Wnt_signaling
Notch_signaling
Contrast
Org+BMDM 1K
Org+BMDM 5K
Org+TNFα
Org+BMDM 1K+TNFα
Org+BMDM 5K+TNFα
Baseline
vs Organoid (untreated)
vs Organoid + TNFa
vs Macrophage (untreated)
vs Macrophage + TNFa
Log2(FC)
40
20
0
-20
-40
e
Yap1
Wwtr1
Tead1
Tead2
Tead3
Tead4
Lats1
Lats2
Stk3
Stk4
Sav1
Mob1a
Mob1b
Nf2
Amotl1
Amotl2
Ctgf
Cyr61
Birc5
Vil1
Alpi
Sis
Epcam
Anpep
Mep1a
Lct
Maoa
Apoa1
Apoa4
Apob
Fabp1
Fabp2
Slc5a1
Slc15a1
Slc2a5
Slc7a8
Muc2
Muc3
Muc13
Tff3
Agr2
Fcgbp
Clca1
Reg4
Spdef
Klf4
Gfi1
Lyz1
Lyz2
Defa3
Defa5
Defa17
Defa21
Defa22
Mmp7
Pla2g2a
Ang4
Itln1
Reg3g
Reg3b
Chga
Chgb
Gcg
Cck
Gip
Sct
Sst
Nts
Ghrl
Neurod1
Pax4
Pax6
Panel
Hippo_YAP
Enterocyte
Goblet
Paneth
Enteroendocrine
Contrast
Org+BMDM 1K
Org+BMDM 5K
Org+TNFα
Org+BMDM 1K+TNFα
Org+BMDM 5K+TNFα
Baseline
vs Organoid (untreated)
vs Organoid + TNFa
vs Macrophage (untreated)
vs Macrophage + TNFa
Log2(FC)
40
20
0
-20
-40

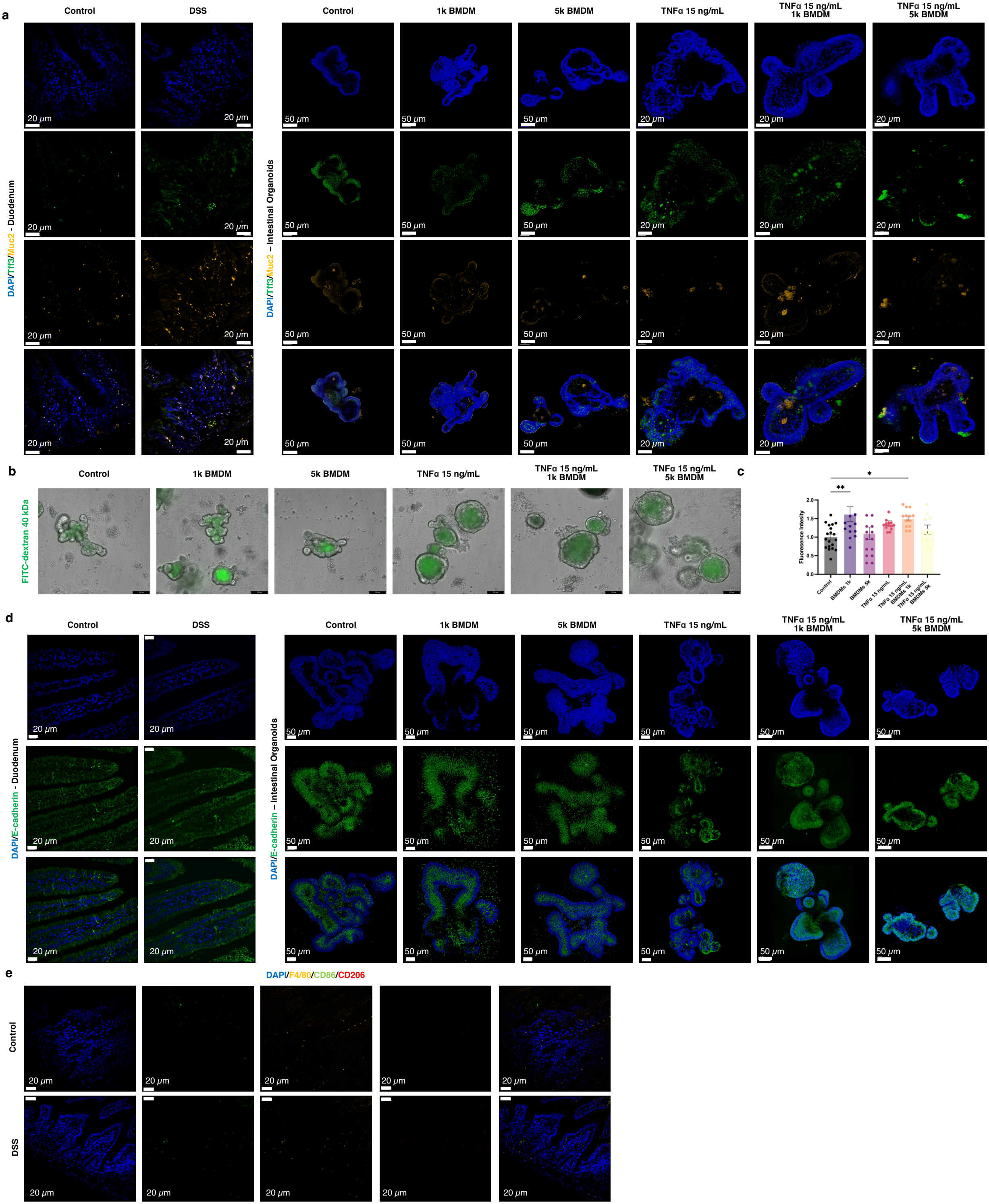

a
Control
DSS
Control
1k BMDM
5k BMDM
TNFα 15 ng/mL
TNFα 15 ng/mL 1k BMDM
TNFα 15 ng/mL 5k BMDM
DAPI/Tff3/Muc2 - Duodenum
DAPI/Tff3/Muc2 – Intestinal Organoids
20 μm
50 μm
b
Control
1k BMDM
5k BMDM
TNFα 15 ng/mL
TNFα 15 ng/mL 1k BMDM
TNFα 15 ng/mL 5k BMDM
FITC-dextran 40 kDa
c
Fluorescence Intesity
2.0
1.5
1.0
0.5
0.0
**
*
Control
BMDMs 1k
BMDMs 5k
TNFα 15 ng/mL
TNFα 15 ng/mL BMDMs 1k
TNFα 15 ng/mL BMDMs 5k
d
Control
DSS
Control
1k BMDM
5k BMDM
TNFα 15 ng/mL
TNFα 15 ng/mL 1k BMDM
TNFα 15 ng/mL 5k BMDM
DAPI/E-cadherin - Duodenum
DAPI/E-cadherin – Intestinal Organoids
20 μm
50 μm
e
DAPI/F4/80/CD86/CD206
Control
DSS
20 μm

Supplementary Figure Legends

Supplementary Figure 1. (a) Representative phase-contrast brightfield images showing morphological changes of L929 fibroblasts during conditioned medium production at Days 3, 5, 7, 10, and 14. Scale bars: 100 µm. (b) Quantification of M-CSF concentrations in L929-conditioned medium by ELISA across time points (Days 0, 3, 5, 7, 10, and 14; n = 3 independent batches). (c) Representative brightfield images of intestinal organoids exposed to increasing proportions of BMDM-conditioned medium (10–50%) mixed with intestinal organoid medium. Scale bars: 100 µm. (d) Quantification of organoid area, diameter, and circularity following exposure to increasing BMDM-conditioned medium concentrations, and ELISA quantification of Il-1β and Il-6 levels across conditioned medium conditions. (e) Representative brightfield images of intestinal organoids co-cultured with 1k or 5k BMDMs in a Transwell-based indirect co-culture configuration, showing organoid morphology at the bottom of the Transwell and within the Matrigel dome. Scale bars: 100 µm. (f) Schematic overview of the co-culture following organoid subculture experimental workflow. (g) Representative brightfield images and quantification of organoid diameter and circularity following co-culture with 1k or 5k BMDMs after organoid passaging at day 3. Scale bars: 100 µm. (h) Representative brightfield images of intestinal organoids co-cultured with high BMDM densities (10k, 25k, 50k, 75k, and 100k BMDMs/well) demonstrating progressive induction of spheroid-like morphology with increasing macrophage density. Scale bars: 100 µm. Data: mean ± SEM. Statistics: one-way ANOVA with Tukey's post-hoc test. *p < 0.05, **p < 0.01, ***p < 0.001, ****p < 0.0001.*

Supplementary Figure 2. (a) Representative immunofluorescence images showing raw channel and merged images of BMDMs stained for DAPI, F4/80, CD86, and CD206 under DMEM+10% FBS, DMEM+10% FBS+20% L929 CM, and IntestiCult conditions. Scale bars: 100 µm. (b) Representative flow cytometry dot plots showing F4/80 vs CD45 (upper panels) and CD86 vs CD206 (lower panels) distribution in BMDMs under DMEM+10% FBS, DMEM+10% FBS+20% L929 CM, IntestiCult, DMEM+10% FBS+20% L929 CM+TNFα, and IntestiCult+TNFα conditions. (c) Quantification of F4/80^{+} macrophage frequency and CD86^{+}CD206^{-}, CD86^{+}CD206^{+}, CD86^{-}CD206^{+}, and CD86^{-}CD206^{-} subpopulations across all conditions (n = 3 independent experiments). (d) Representative immunofluorescence images of intestinal organoids co-cultured with 1k or 5k BMDMs stained for DAPI and Iba-1, showing macrophage localization in proximity to and within organoid structures. Scale bars: 30 µm. (e) Representative immunofluorescence images of histological sections of co-cultured organoids stained for DAPI, F4/80, CD86, and CD206 under 1k and 5k BMDM conditions, showing individual raw channels and merged image. Scale bars: 20 µm. Representative images from 5 independent experiments. Data: mean ± SEM. Statistics: one-way ANOVA with Tukey's post-hoc test. *p < 0.05, **p < 0.01, ***p < 0.001, ****p < 0.0001.*

Supplementary Figure 3. (a) Representative flow cytometry dot plots showing F4/80 vs CD45 distribution in 1k and 5k BMDM co-culture conditions, with macrophage gate indicated. (b) Quantification of F4/80^{+} macrophage frequency as percentage of total live cells in 1k and 5k co-culture conditions (n = 3 independent experiments). (c) Complete gating strategy for macrophage identification showing sequential gates: FSC-A vs SSC-A (cell gate), FSC-H vs FSC-A (singlet discrimination), SSC-A vs Live/DEAD (viability gate), SSC-A vs CD45 (CD45^{+} gate), F4/80 vs

CD45 (macrophage gate), and CD86 vs CD206 (polarization analysis) within the CD45$^+$F4/80$^+$ population. (d) Complete gating strategy for epithelial and immune cell identification in co-culture conditions showing sequential gates: FSC-A vs SSC-A (cell gate), FSC-H vs FSC-A (singlet discrimination), SSC-A vs Live/DEAD (viability gate), SSC-A vs CD45 (immune cell identification), and SSC-A vs EpCam (epithelial cell identification) within the CD45$^-$ population. Data: mean ± SEM. Statistics: unpaired Student's t-test. *p < 0.05, **p < 0.01, ***p < 0.001, ****p < 0.0001.*

Supplementary Figure 4. (a) Representative high-resolution immunofluorescence images showing individual raw channels and merged image of intestinal organoids under control, 1k, and 5k BMDM conditions stained for Lgr5, Ki67, and DAPI. Scale bars: 40 µm. (b) Representative high-resolution immunofluorescence images showing individual raw channels and merged image of organoids stained for CD24, Lyz, and DAPI under control, 1k, and 5k BMDM conditions. Scale bars: 40 µm. (c) Representative high-resolution immunofluorescence images showing individual raw channels and merged image of organoids stained for Sox9 and DAPI under control, 1k, and 5k BMDM conditions. Scale bars: 30 µm. (d) Heatmap showing Log2 fold change of intestinal stem cell markers, WNT signaling, and Notch signaling-associated genes in 1k and 5k BMDM co-culture conditions compared against untreated organoid and macrophage monoculture baselines (n = 4 samples/group). (e) Heatmap showing Log2 fold change of Hippo-YAP, enterocyte, goblet, Paneth, and enteroendocrine lineage-associated genes in 1k and 5k BMDM co-culture conditions compared against untreated organoid and macrophage monoculture baselines (n = 4 samples/group). Data are represented as Log2(FC) relative to indicated baselines.

Supplementary Figure 5. (a) Representative immunofluorescence images of intestinal organoids co-cultured with 1k or 5k BMDMs under TNFα (15 ng/mL) stimulation, stained for DAPI and Iba-1, showing macrophage localization in proximity to and within organoid structures. Scale bars: 30 µm. (b) Representative immunofluorescence images of histological sections of TNFα-treated co-cultured organoids stained for DAPI, F4/80, CD86, and CD206 under 1k and 5k BMDM conditions, showing individual raw channels and merged image. Scale bars: 20 µm. (c) Representative flow cytometry dot plots showing F4/80 vs CD45 distribution in 1k and 5k BMDM co-culture conditions following TNFα stimulation, with macrophage gate indicated. (d) Quantification of F4/80$^+$ macrophage frequency as percentage of total live cells across 1k and 5k BMDM conditions with and without TNFα stimulation (n = 3 independent experiments). Representative images from 5 independent experiments. Data: mean ± SEM. Statistics: one-way ANOVA with Tukey's post-hoc test. *p < 0.05, **p < 0.01, ***p < 0.001, ****p < 0.0001.*

Supplementary Figure 6. (a) Representative high-resolution immunofluorescence images showing individual raw channels and merged image of intestinal organoids exposed to TNFα (15 ng/mL) alone or in combination with 1k or 5k BMDMs, stained for Lgr5, Ki67, and DAPI. Scale bars: 40–50 µm. (b) Representative high-resolution immunofluorescence images showing individual raw channels and merged image of TNFα-treated organoids stained for CD24, Lyz, and DAPI under TNFα alone, TNFα+1k, and TNFα+5k BMDM conditions. Scale bars: 50 µm. (c) Representative high-resolution immunofluorescence images showing individual raw channels and merged image of TNFα-treated

organoids stained for Sox9 and DAPI. Scale bars: 30–50 µm. (d) Heatmap showing Log2 fold change of intestinal stem cell markers, WNT signaling, and Notch signaling-associated genes across TNFα-treated 1k and 5k BMDM co-culture conditions compared against untreated and TNFα-treated organoid and macrophage monoculture baselines (n = 4 samples/group). (e) Heatmap showing Log2 fold change of Hippo-YAP, enterocyte, goblet, Paneth, and enteroendocrine lineage-associated genes across TNFα-treated co-culture conditions compared against untreated and TNFα-treated organoid and macrophage monoculture baselines (n = 4 samples/group). Data are represented as Log2(FC) relative to indicated baselines.

Supplementary Figure 7. (a) Representative immunofluorescence images showing individual raw channels and merged images of duodenal tissue sections from control and DSS-treated mice (left panels) and intestinal organoids across all experimental conditions (right panels), stained for DAPI, Tff3, and Muc2. Scale bars: 20 µm (duodenum) and 20–50 µm (organoids). (b) Representative brightfield and fluorescence overlay images of FITC–dextran (40 kDa) permeability assay in intestinal organoids under control, 1k BMDM, 5k BMDM, TNFα, TNFα+1k BMDM, and TNFα+5k BMDM conditions. Scale bars: 100 µm. (c) Quantification of luminal fluorescence intensity normalized to organoid area across experimental conditions (n ≥ 10 organoids/condition). (d) Representative immunofluorescence images showing individual raw channels and merged images of duodenal tissue sections from control and DSS-treated mice (left panels) and intestinal organoids across all experimental conditions (right panels), stained for DAPI and E-cadherin. Scale bars: 20 µm (duodenum) and 50 µm (organoids). (e) Representative immunofluorescence images of duodenal tissue sections from control and DSS-treated mice stained for DAPI, F4/80, CD86, and CD206, showing individual raw channels and merged image. Scale bars: 20 µm. Representative images from 5 independent experiments. Data: mean ± SEM. Statistics: one-way ANOVA with Tukey's post-hoc test. *$p < 0.05$, **$p < 0.01$, ***$p < 0.001$, ***$p < 0.0001$.

Supplementary table 1. Western Blot antibody list

Primary Antibody	Dilution concentration	Catalog number
**B-actin**	1:1000	inVitrogen #15G5A11/E2
**B-catenin**	1:1000	Cell Signaling #37447
**Bmi1**	1:5000	Abcam #ab254253
**Cleaved-caspase3**	1:1000	Cell Signaling #9664
**Lgr5**	1:250	BD Pharmingen #562731
**Lysozyme**	1:1000	Cell Signaling #60487
**Notch1**	1:1000	Cell Signaling #3608
**Phospho-SAPK/JNK**	1:1000	Cell Signaling #9251
**Sox9**	1:1000	Abcam #ab185230
**Tff3**	1:1000	inVitrogen #PA5-21081
**Wnt3a**	1:1000	Cell Signaling #2721
**YAP/TAZ**	1:1000	Cell Signaling #8418

Secondary Antibody	Dilution concentration	Catalog number
**Anti-mouse**	1:1000	Cell Signaling #7076P2
**Anti-rabbit**	1:1000	Cell Signaling #7074P2
**Anti-rat**	1:10000	ThermoScientific #31470

Supplementary table 2. Immunofluorescence antibody list

Primary Antibody	Dilution concentration	Catalog number
**APC anti-mouse CD206**	1:50	clone C068C2, Cat#141708, BioLegend
**CD24**	1:250	inVitrogen #14-00242-82
**FITC anti-mouse CD86**	1:50	clone GL-1, Cat# E-AB-F0994UC, Elabscience
**Ki67**	1:1000	Abcam #16667
**Lgr5**	1:250	BD Pharmingen #562731
**Lysozyme**	1:250	Cell Signaling #60487
**Muc2**	1:250	Novusbio #996/1
**PE anti-mouse F4/80**	1:50	clone BM8, Cat# 123110, BioLegend
**Sox9**	1:250	Abcam #ab185230
**Tff3**	1:200	inVitrogen #PA5-21081

Secondary Antibody	Dilution concentration	Catalog number
**Anti-mouse Alexa594**	1:500	inVitrogen #A21203
**Anti-rabbit Alexa488**	1:500	inVitrogen #A11008
**Anti-rabbit Alexa594**	1:500	inVitrogen #A11012
**Anti-rat Alexa488**	1:500	inVitrogen #A11006

Supplementary table 3. Flow cytometry antibody list

Antibody	Dilution Concentration	Catalog number
**APC anti-mouse CD206**	1:200	BioLegend, clone C068C2, # 141708
**FITC anti-mouse CD86**	1:50	Elabscience, clone GL-1, #E-AB-F0994UC
**LIVE/DEAD™ Fixable Aqua Dead Cell Stain Kit**	1:1000	Invitrogen, #L34966
**PE anti-mouse CD326**	1:200	Invitrogen, clone G8.8, #12-5791-82
**PE anti-mouse F4/80**	1:200	BioLegend, clone BM8, #123110
**PerCP anti-mouse CD45**	1:200	BioLegend, clone 30-F11, #103129
